# A new conceptual framework for managing hamstring injury risk in soccer – implementing a data-informed approach: a narrative review

**DOI:** 10.5114/biolsport.2025.151660

**Published:** 2025-09-16

**Authors:** Guglielmo Pillitteri, Filipe Manuel Clemente, Marco Petrucci, Hugo Sarmento, Antonio Figueiredo, Tindaro Bongiovanni, Antonino Bianco, Giuseppe Battaglia, Tim J. Gabbett

**Affiliations:** 1 Sport and Exercise Sciences Research Unit, Department of Psychology, Educational Science and Human Movement, University of Palermo, Palermo, Italy; 2 High-Performance Unit, Palermo FC, Italy; 3 Escola Superior Desporto e Lazer, Instituto Politécnico de Viana do Castelo, Rua Escola Industrial e Comercial de Nun’Álvares, 4900-347 Viana do Castelo, Portugal; 4 Gdansk University of Physical Education and Sport, 80-336 Gdańsk, Poland; 5 Sport Physical Activity and Health Research & Innovation Center, Viana do Castelo, Portugal; 6 Research Unit for Sport and Physical Activity (CIDAF), Faculty of Sport Sciences and Physical Education, University of Coimbra, Coimbra, Portugal; 7 Department of Biomedical and Neuromotor Sciences (DIBINEM), Alma Mater Studiorum—University of Bologna, Bologna, Italy; 8 Gabbett Performance Solutions, Brisbane, QLD, Australia

**Keywords:** Injury, Soccer, Hamstring, Framework, Data-informed

## Abstract

The multifactorial and complex nature of athletic injuries complicates the establishment of clear cause-and-effect relationships, making it challenging to pinpoint precise injury aetiology in the operational field. Research has explored the connection between injuries and training load (TL), identifying an association between high external loads (EL)—such as high-speed running (HSR) and sprinting—and non-contact hamstring injuries. Despite this, injury prevention remains largely ineffective, possibly reflecting a gap between scientific knowledge and practical application, as well as cognitive biases in translating research into real-world scenarios. This paper aims to: 1) summarize key findings on non-contact injuries in soccer, focusing on risk factors and the TL–injury relationship; 2) describe major conceptual frameworks and highlight cognitive biases when attributing injuries exclusively to TL; 3) propose new conceptual frameworks for non-contact injuries, with a primary focus on hamstring injuries. Using a qualitative approach, we present a multilevel causation framework that underscores the significant—but not exclusive—role of sprinting and TL in injury occurrence. We introduce two key concepts: acute mechanical load tissue exposure (AMLTE), referring to the peak acute mechanical stress a muscle can tolerate, and chronic muscle tolerance to load (CMTTL), representing the capacity to endure load over time. We aimed to support a data-informed approach to injury risk management and TL monitoring in daily practice. While we stress that TL and sprinting are not the sole causes of injury, we assert their relevance within a multifactorial model. This framework may assist professionals in developing more effective training strategies and injury prevention practices.

## INTRODUCTION

Despite significant efforts from the scientific community, sporting injuries, particularly hamstring injuries, have increased in the last two decades [1, 2]. This inevitably, and justifiably, results in the questioning of the efficacy of contemporary injury prevention and rehabilitation strategies. Accordingly, it is important that the scientific community critically assesses its approaches in this area [3]. Within the literature, various factors have been associated with injury [4–11]. Typically, these research approaches have commonly centred on singular risk factors, with a large quantity of research focusing specifically on training load (TL) [12, 13]. Here, there is a risk that the lens adopted is too narrow, and too much emphasis has been placed on too few variables, such as singular training load metrics, as ‘the cause’ of injury [14]. This has likely contributed to incomplete understandings of injury prevention that fails to appropriately capture its multifactorial nature [9, 10, 15, 16].

Although several factors are related to injury [17], clear causeand-effect relationships between many of these factors and injury occurrence have not been established [8, 18–20]. A relevant contribution highlighting a lack of causal understanding in athletic injury research has been offered by Kalkhoven, who outlined the general absence and lack of utilisation of appropriate causal inference structures and tools (such as frameworks, models and causal directed acyclic graphs (DAGs)) to guide causal inferences within athletic injury research [20]. Despite these limitations, and while not necessarily reflective of causation, there have been many studies that have investigated the relationship between injuries and TL, finding an association between high EL, such as HSR, sprinting and noncontact hamstring injury [21–23]. In the same way, studies have shown that rapid increases in both EL and internal load, based on the acute chronic workload ratio (ACWR) [24], are associated with injuries even if other fundamental factors such as moderators (i.e. internal characteristics such as strength and aerobic fitness that may increase or decrease injury risk at given workloads) and mediators (i.e. factors that increase the risk of injury, such as neuromuscular fatigue) have not been considered [25]. Consequently, care must be taken when applying this information in practice.

Despite these limitations, some studies have provided valuable contributions focusing on specific training-related factors which could play an important role in optimizing TL [26] and managing injury [6, 25, 27, 28]. Indeed, the large amount of data collected by electronic performance and tracking devices such as Global Positioning System (GPS) technology has assisted decision-making using a data-informed approach, which also has included attempts to predict injury through machine learning (ML) approaches [29].

For example, Rossi and colleagues [30] predicted injuries in soccer players via a multi-dimensional analysis in which only GPS (i.e., external load) measurements were considered, while Vallance et al. [31]. used both internal and EL to predict injury. While promising results could be offered by prediction studies in the future, Bullock et al. strongly questioned the quality of existing prediction models, reporting that 98% of sport musculoskeletal injury prediction models (and 79% of studies) were rated as high risk of bias [32]. This may potentially explain the large amount of conflicting results within the literature pertaining to the relationships between TL and injury [8, 19, 20, 33]. Indeed, Jasper et al. [34] reported that the evidence relating both external and internal load to injuries is scarce, suggesting that daily monitoring of TL along with the individual player’s characteristics allows a better understanding of the relationship between TL, physical fitness, player’s condition and injury risk (IR).

In light of this, Gabbett [6] proposed that effective TL management, which entails avoiding TL spikes while simultaneously enhancing chronic load, may improve players’ fitness and reduce injury rates. Indeed, in cricket fast bowlers, players who accrued greater chronic loads experienced lower injury risk than players with lower chronic loads [35]. Although these findings require further research confirmation, it has been indicated that high-intensity metrics might be most suitable for detecting injury risk in athletes. This is particularly evident for hamstring strain injuries, with HSR and sprinting loads associated with these events [5, 15, 20, 36].

Although studies to date have contributed to the understanding of training load-injury relationships in various contexts [14], unfortunately, no study satisfies the criteria for establishing an appropriate cause-and-effect relationship. Indeed, randomized controlled trials (RCTs) investigating the TL and injury relationship are scarce [20, 37]. Furthermore, the majority of studies in this area are retrospective investigations and observational, which are particularly susceptible to various forms of bias, such as confounding—which occurs when a third variable, known as a confounder, affects both the independent variable (the cause or treatment being studied) and the dependent variable (the outcome or effect being measured) [20], leading to a spurious (non-causal) associations between variables. Accordingly, until appropriate causal inference tools, processes and methods are developed and adopted, which is a complex undertaking due to the multifactorial nature of injury, it is only possible to detect associations or make predictions from such data, and claims of causation should be avoided (Kalkhoven 2024) [20, 30, 38]. This emphasises the lack of an identified cause-effect relation between the factors analysed and the onset of injuries [8, 20].

In practical settings, due to the complex nature of athletic injuries, when a single factor, such as training load, is attributed as the exclusive cause to the occurrence of an injury, a cognitive bias may drive reasoning at the expense of more logical approaches based on accurate knowledge of the underlying injury mechanisms. These risks misunderstand the “real” role of TL in the injury causation and injury reduction process [4, 9, 15, 20], leading to poor training load management strategies. Instead, a more logical approach might be to understand the “weight” of each risk factor implicated in the onset of injuries and how these risk factors are interconnected [4], thereby avoiding rash judgments and poor decision-making based on cognitive biases [20, 39]. To avoid these issues, it is important to properly conceptualise the problem of athletic injury occurrence through the use of conceptual frameworks [4, 9, 20]. This will assist with both defining the problem of injury occurrence, laying bare any causal assumptions, and understanding the limitations in the measures that underpin decision-making processes [20].

While Kalkhoven [20] provided a useful theoretical contribution and in depth understanding relating to the need for implementing causal inference approaches to injury research, suggesting the use of frameworks and appropriate DAGs, Gabbett *et al.* provided a practical framework on training monitoring and athlete management [11, 25, 40]. These authors suggested that a combination of EL, internal load, perceptual well-being, and readiness to train/compete data delivers more meaningful individual training prescriptions compared to data (data-driven approach) derived from any single athlete monitoring tool, ultimately leading to a more holistic injury prevention strategy [40, 41].

Based on the previous consideration, two concepts should be kept in mind. The first concerns the awareness that although TL may be implicated in injury occurrences [9, 15, 20], current measures may not be sensitive enough to provide precise or reliable predictions of injury risk necessary to justify a data-driven approach to managing athletic injury risk management using these data. No study has ever demonstrated, with the appropriate methods, a causal association of TL with injury [20], while the emphasis on a single factor for predicting athlete injuries (TL and any EL variables such as sprinting included) is likely too reductionist to yield reliable predictions. The second concerns the on-field practice of training load management, which is the main factor that sports scientists can influence on a dayto-day basis. In this context, a data-informed approach that is carefully integrated with the goals of the athlete and the organisation is recommended to optimize training prescription (e.g. optimal adaptation, enhancement of physical performance, and potentially injury prevention). This will help minimise potential conflicts among relevant stakeholders, e.g., athletes, staff, organisation, regarding inappropriate restrictions of training load that overreach in their interference with training processes. In this approach, using training load to manage injury should be secondary to, and should not undermine in a conflict-inducing manner, the performance goals of the athlete or organisation [42] [43, 44]. Accordingly, when decisions are made in relation to restricting training load, a holistic approach should be adopted where such decisions are not made using an isolated data-driven approach, i.e., by training load data alone, but should be carefully weighted against the training and performance requirements of the athlete and organisation.

Since confusion, misinterpretations and cognitive bias may still be present in the real world, as highlighted by the small effect of injury prevention strategies [2], our conceptual framework will explore injuries that occur in soccer, focusing on the main role of TL. Hence, the aims of this paper are to 1) summarize the main findings on noncontact injury in soccer, reporting the most important risk factors for hamstring injuries, including the relationship between TL and injury; and 2) describe a conceptual framework of soccer injuries, highlighting a possible cognitive bias when practitioners attribute a single cause, such as training load, to injuries. Finally, we present a qualitative approach through a new conceptual framework on noncontact soccer injuries by including an example from high performance sport where a data-informed approach is used to support noncontact hamstring injury prevention strategies.

To achieve our objectives, this article is structured as follows: Section two delves into the prevalent incidence of injuries in soccer, specifically hamstring strains, elucidating advances in causal understanding and emphasising the critical need for accurate injury reporting and robust prevention strategies. It highlights the complex biomechanics of hamstring function during activities such as HSR and sprinting. Section three explores cognitive bias as it relates to injury and training load, stressing the importance of recognizing injuries’ multifactorial nature and the pitfalls of oversimplified causal explanations. Section four critically reviews conceptual frameworks on sports injuries, emphasizing their multifaceted origins and cautioning against attributing injuries solely to TL. Section five examines conceptual frameworks on training load and injury in soccer, emphasizing the intricate interplay between workload and injury risk. It describes the necessity of optimizing performance while minimizing injury risk through balanced training intensity and duration. Finally, section six introduces a data-informed approach to load management in soccer, demonstrating how integrating the monitoring of external and internal loads with applied physiology and training methodology can support training prescriptions, and careful consideration should be taken when implementing load managements to reduce injury risk when it is in conflict with the training and performance goals of the athlete.

### What about non-contact injuries in soccer?

In soccer, hamstring injuries are prevalent and costly, impacting both player health and club finances. Epidemiological studies indicate high injury rates, particularly during matches and among professional male players. The focus on hamstring injuries centres on their biomechanical causes during HSR and sprinting, where the muscle’s role as an eccentric brake is crucial in decelerating knee extension. Despite differing theories on the exact injury mechanisms—whether during late swing or early stance phases—research underscores the need for targeted prevention strategies [45]. Factors such as insufficient eccentric strength and inadequate exposure to HSR during training contribute significantly to injury risk, necessitating precise training-to-match ratios and tailored exercises to enhance muscle capacity and mitigate injuries. Understanding these biomechanical complexities not only informs injury prevention but also underscores the complex interplay between TL, muscle function, and injury occurrence in soccer.

In 2006, Fuller et al. published a consensus statement [46] addressing soccer injuries. This was prompted by the variety of definitions and methodologies employed in studies, resulting in conflicting results, confusion, and challenges in comparing studies. In an updated version [47], the authors provided recommendations on how injury incidences should be reported to mitigate methodological discrepancies when comparing studies [46]. For example, the authors suggested accurately reporting definitions of injury, recurrent injury, severity, training and match exposures, location, type, diagnosis, and causation [47].

Soccer injuries have been extensively studied, as they have significant negative repercussions for the health of players and the financial burden of clubs [1, 48]. Indeed, the mean cost of an injured player in a professional team has been reported to be €500,000/ month [49]. Readers are further encouraged to consult the recent review by Pulici et al. [50] on injury burden for a more comprehensive and updated perspective on the economic impact.

Epidemiological studies have reported that soccer results in high injury incidences at the professional, amateur, and youth levels [51–54]. Overall, the incidence of injuries in male professional soccer players is 8.1 injuries/1,000 hours of exposure [55]. At both the professional and amateur levels, the incidence of injuries is higher in matches than in training sessions, with the lower extremities having the highest incidence rates [55, 56].

Specifically, hamstring strain injury (HSI) is the most prevalent non-contact injury reported in professional male soccer (12–14% of all injuries [1]), accounting for 37% of all muscle injuries sustained [48, 57, 58]. Furthermore, the reinjury rate of HSIs ranges from 16 to 60% [57]. In contrast, anterior cruciate ligament (ACL) injuries are considered among the most severe, with a time loss of 185–256 days per injury [58, 59]. Ekstrand et al. [52] reported that players can have a mean of 14 competition days lost per injury (range: 1–128 days for muscle injuries), severely harming team performance and imposing a financial burden [58, 60] on the players and their clubs [60].

#### Knowledge about hamstring injury aetiology

The high incidence of injuries can be attributed to numerous factors (multifactorial aetiology [10, 16, 45]), including the high intensity of play, which has increased over the years, and the large number of matches played during the season. Given the negative impacts of injuries on sports teams, there is a pressing need to enhance the assessment and multi-analysis capabilities to pinpoint the primary factors associated with injuries. This will aid practitioners in identifying strategies to mitigate injury risk through interventions and data-informed practices.

Although several studies have focused on identifying the main risk factors of hamstring injuries [61–64] and implementing effective prevention and rehabilitation strategies [65–69], a consensus on hamstring injury mechanisms has never been established. Specifically, key risk factors for hamstring injuries include older age, a history of previous hamstring strain injuries, and neuromuscular fatigue, as highlighted in a recent systematic review [70]. For further insight into hamstring injury risk factors, readers are referred to recent systematic reviews such as Green et al. [70]. Two main hamstring injury mechanisms related to running have been reported in the literature [71]. The first is that a hamstring injury is more likely when experiencing the highest load during active lengthening (simultaneous hip flexion and knee extension) observed in the late swing phase (which commences as the hip reaches peak flexion at approximately 100° and then starts to extend in preparation for the foot strike) [71] of the running gait cycle [72, 73]. Conversely, Mann et al. [74] considered that the primary injury mechanism is the initial stance phase (which begins at foot strike, where the hip extends while the knee flexes) [71], because it involves large forces working in opposing directions as the body is pushed forward over the touchdown point.

Although most studies on hamstring injury during running have reported high rates of injuries during the late swing phase [75, 76], the stance phase has also been considered critical for this kind of injury due to considerable eccentric loading. Certainly, the biceps femoris long head (BFlh) shows greater force during the stance phase, while the semitendinosus (ST) shows greater force than the BFlh during the swing phase. As the BFlh is more susceptible to injury during running than the ST, hamstring injuries are more likely during the stance phase [77], while several studies have reported that hamstring injuries occurs during the late swing phase of sprinting [76, 78–80].

Askling et al. reported that hamstring injuries can occur during either HSR or stretching movements [67]. An injury caused by the first type of movement affects the BFlh and shows a lower severity and a shorter recovery time than an injury caused by stretching movements, which commonly involve the semimembranosus [66, 77, 81].

Of note, fatigue reduces hamstring eccentric strength, thereby increasing the risk of injury [82, 83], and reinjury is associated with lower hamstring strength endurance [84]. Given the relevant biomechanical implications of HSR activities for hamstring injuries [85], studies have implemented prevention and rehabilitation strategies targeting these activities. One study carried out by Aiello et al. [86] reported that 16 of 17 hamstring injuries occurred when the players were accelerating to or decelerating from a speed of > 25 km/h (i.e. HSR, very HSR, or sprinting) [87]. Specifically, players were running for (median and interquartile range) 16.75 m (8.42–26.65 m) and achieved a peak speed of 29.28 km/h (26.61–31.13 km/h), which corresponded to 87.55% of players’ maximal speed (78.5–89.75%). Moreover, injuries occurred whilst players were in a position of hip flexion and knee extension (i.e., the late swing phase) [97] experiencing high eccentric hamstring loading, in accordance with previous studies [88].

Given the effect of HSR on non-contact injuries [89], periodized exposure to both HSR and sprinting within the microcycle of a soccer season could help players tolerate the demands of competition, while minimizing injury risk. Accordingly, practitioners should seek an optimal training-to-match HSR ratio even though high variability exists due to different training methodologies among teams. For example, Clemente et al. [90] analysed the training-match ratio (calculated based on total distance, player load and total number of high (> 3 m · s^−2^) accelerations and decelerations) and found that, on average, training sessions represent 1.8 times the effort expended in a single match. However, this Figure can vary (ranging from 0.6 to 1.8) significantly depending on the player’s position and the number of training sessions per week [91, 92].

The underexposure of players to HSR during training may significantly impact their ability to adapt to the demands of a match. Buchheit et al. [5] suggested an association between accumulated HSR and sprinting distance during the training days over the microcycle and match injury occurrence. The authors [5] found no match hamstring injuries when players were exposed to running bouts at > 90% maximal sprinting speed (i.e. a five-day microcycle) and > 95% maximal sprinting speed (i.e. three-, five- and six-day microcycles) during training sessions leading into matches. However, injuries still occurred during 85% of the microcycle when there were no or low volumes of high-velocity speed exposures (> 85 or > 90%). These results suggest the paramount role of peak speed in adapting the hamstring muscles to withstand match demands. A contribution on this topic has been offered by Buchheit [21, 22, 93], concerning the training microcycle programming based on scientific evidence aiming to “informing” the experts to support make decisions. Specifically, the author explored the management of post-match recovery and compensation training, suggesting the post-match (MD+1) need to compensate for high intensity and sprint training to maintain the performance of substitutes and reduce the risk of injury [93–95]. A paramount factor concerns the highspeed peaks, as a strategy to reduce the risk of injury. Indeed, the importance of training at near-maximum speed (> 95%) at MD-2 may be associated with a reduction in injury rates [21, 22].

Physiological aspects related to soccer players and the references values derived from match-demand knowledge should be considered when training programme design is required. For example, professional players cover total distances ranging between 10 and 13 km, of which around 900 m and 250–300 m are covered while HSR (speed ranging from 19.8 km · h^−1^ to 25.2 km · h^−1^) and sprinting (speed ≥ 25.2 km · h^−1^), respectively [89, 96]. The importance of HSR and sprinting distance has been recently discussed in a systematic review [89]. Specifically, during official matches, HSR and sprint running distances ranged from 618 to 1,001 m and 153–295 m, respectively, in professional male soccer players [89]. These values should be borne in mind to optimize soccer training since they are strongly implicated in hamstring stress [15, 67, 89, 93]. In this way, understanding hamstring functioning during HSR and sprinting considering their implications for hamstring injury could help practitioners to consider appropriate prevention strategies to mitigate injury risk.

#### Hamstring functioning during high-speed running and sprinting and its injury implications

In the soccer context, most hamstring injuries occur when players are sprinting at above 80% of maximum speed [86], highlighting the strong relationship between high-intensity running and hamstring loading.

New theories—such as the spring-like hamstring phenomenon proposed by Van Hooren and Bosch [98, 99] on hamstring functioning during sprints involving the eccentric lengthening of the hamstring muscles in the late swing phase—have been questioned. Kalkhoven et al. [15] provided a detailed conceptual exploration of hamstring muscle–tendon functioning during the late swing phase of sprinting. To understand the complex hamstring functioning during sprinting, Kalkhoven et al. [11] comprehensively examined different models of myotendinous unit (MTU) functioning during locomotion [15].

Two main models of MTU functioning during locomotion have been reported in the literature: an efficiency model and a power model [100, 101]. The efficiency model relates to spring-driven MTU behaviour by which muscles act mainly as mechanical struts and perform minimal work while the tendons work by storing and releasing elastic energy to improve locomotor efficiency [100]. Conversely, the motor-driven power model of MTU functioning considers muscle actuators and active muscle work as a process by which muscle fibres usually experience large excursions, especially during high-intensity circumstances, with the muscle fibres shortening to produce power [100].

The spring-driven model saves metabolic energy during sprints, as the muscle (contractile element) works isometrically while the tendon performs all the mechanical work. However, some questions have been raised in the literature. Studies have found no difference in the metabolic cost of force production between muscular stretch– shortening cycles (during which the muscle lengthens while active) and isometric contractions [102]. Moreover, even though the motordriven system is predominantly active during high-intensity actions, tendons act as transmitters of muscle contractile force to the origininsertion points for the generation of limb movements instead of storing and releasing elastic energy [101].

Spring-driven and motor-driven models are not exclusive and can be used according to the task demands, highlighting that muscles act eccentrically under both conditions [103], requiring force production, energy absorption, or utilization of the stretch–shortening cycle.

Accordingly, the isometric theory should be considered with caution. It should be kept in mind that hamstring muscles adopt a model of functioning including both muscle actuators and active muscle lengthening. Moreover, the amount of eccentric work leading to muscle damage has been related to hamstring injuries during HSR [104].

Conversely, Van Hooren and Bosch’s [98, 99] theory indicates that the hamstring muscles passively lengthen during the initial and mid-swing phases of sprinting due to muscle slack during the delay between the start of contractile element activation and series elastic element recoil [105]. However, the existence of muscle slack has been questioned in electromyographic (EMG) and electromechanical studies showing that the hamstrings receive electrical signalling throughout the sprint cycle [15, 106]. Moreover, regarding large knee joint ranges with changes in the MTU length that occur during sprints [80, 104], it has been hypothesized that the hamstring fascicles would be required to actively (eccentrically) lengthen and shorten to complete the sprint cycle.

Considering the kinematic and kinetic perspective of hamstring function during the swing phase of sprinting, studies have reported that, being biarticular (except for the biceps femoris short head), hamstring muscles are stretched during the mid-swing phase as the hip is in a flexed position while the knee is extending [76]. Near the end of the mid-swing phase, an exceptionally high peak knee joint extension velocity (> 1000°/s) has been reported with a consequent rapid deceleration of the knee joint [15]. This has been seen with EMG data leading to the active braking role of the hamstrings, especially in the late swing phase, during which they are stretched to maximum or near-maximum length [76]. As the eccentric role of the hamstring has been widely established, Kalkhoven et al. [15] made valuable recommendations for hamstring-specific training and injury prevention exercise selection.

Researchers claim that the adaptation derived from eccentric hamstring training may reduce the risk of hamstring injury during sprinting activities [107]. Of note, muscle functioning during sprints is derived from the complex interaction between musculoskeletal kinematics and kinetics [108], muscle activation patterns, the neuromechanical regulation of tensions and stiffness [109], and loads applied by the environment. Accordingly, exercises activate the hamstring muscles at a maximum of 60% of the maximal activation exhibited during top-speed sprinting [110]. This results in specific mechanical demands influencing hamstring MTU function during sprinting, including the precise timing and combination of hip and knee joint angles, velocities, accelerations and moments, as well as the magnitude and rate of muscle loading, and the required interplay of muscle and tendon lengths, length changes, and their respective velocities and accelerations. [15]. We invite readers to consult Kalkhoven et al. [15] to delve deeper into the MTU functioning mentioned above.

In summary, the behaviour of the MTU of the hamstring during sprinting is highly specific and finely regulated for this activity. Selecting hamstring exercises in isolation to replicate hamstring functioning during sprinting is questionable despite the adaptive benefits derived from eccentric exercises specific for the hamstrings for athletes called upon to sprint [15]. Therefore, to reduce hamstring injury risk during sprints, practitioners should administer sprint training to enhance the hamstring loading capacity [5]. Understanding the key physiological and biomechanical characteristics underlying hamstring function has shed light on the role of sprinting and HSR management. However, despite the implication of individual factors related to TL such as sprinting, the exclusive causal attribution of load to injury occurrence can be considered a cognitive bias, as we will explore in the next section by following a “psychosociological” perspective [111, 112].

### Cognitive bias behind the exclusive causal attribution of load on injury occurrence: can the causal-effect approach explain the acute injury event?

Several factors, including TL, have been investigated in the literature [8, 9, 14, 34] to fully understand the causal reasons for acute injuries. In the soccer context, practitioners have investigated causal factors that may lead to a non-contact injury, even though the multifactorial aetiology has been widely approved by the scientific community [8, 10, 16]. Put simply, non-contact injuries usually happen during high-stress situations, such as the intensified phase of both official matches and training due to high physical stress and mechanical force that the tissue cannot support [9, 15]. The stress and strain experienced by a particular tissue are determined by the interaction between the force applied to a specific tissue, the mechanical properties of the tissue itself, and its current physiological condition—particularly its fatigue status—which may alter its ability to tolerate mechanical load and increase the risk of injury [9].

Accordingly, the inappropriate “load” administered to a predisposed (i.e. not adapted or weak) player seems highly involved in the occurrence of acute injury [9, 11, 25]. Although a correlation between TL and injury has been found [14], causation has never been confirmed, highlighting the lack of appropriate study design and methodologies (such as RCTs as a gold standard). Indeed, causation requires an association between two variables, but association does not necessarily imply causation [20]. An association between two factors can occur both with and without a causal relationship. Accordingly, it seems inappropriate and illogical to attribute the cause of a given injury to a single factor related exclusively to work load. In the study of systemic pathologies, such as cardiovascular disease [113], identifying the main risk factors may help reduce the onset and incidence of illness. For further knowledge on the need for causal inference on injury, we refer readers to Kalkhoven [20]. The author highlighted the unfeasibility of RCTs in elite real soccer context, suggesting potential strategies that can assist with developing causal understandings of athletic injury such as development and use of frameworks, models and causal DAGs to overcome the “obstacles” and provide value for research pursuits [20].

While Kalkhoven refers to a high scientific perspective providing a useful solution to understand the causative knowledge, according to a “sociopsychological” perspective, the study of injuries should start with logical reasoning (i.e. a high cognitive process) considering the possible factors that interact to determine the onset of injury. For example, a spike in TL could cause an injury in weak players, as reported by Windt and Gabbett [11]. Unfortunately, the study of higher cognitive processes such as thinking, reasoning, decisionmaking, and social cognition has been related to bias, error, and irrationality. It was reported [39] that bad thinking—such as illogical reasoning, trivial decision-making, prejudice and stereotyping—affects people in different contexts. In this way, a cognitive bias, defined as a systematic (not random) error could be the result of a misunderstanding or wrong reasoning when a single factor (including TL and its indicators such as sprinting or HSR) is attributed as the exclusive cause of an injury [8, 9].

Psychologists have reported that individuals’ evaluations of logical arguments are biased by the degree to which the conclusions align with their beliefs [39, 114]. Moreover, previous knowledge and beliefs influence human reasoning, and only a strong effort of conscious deliberative reasoning can overcome this [39, 115]. In the injury field, practitioners have tried to predict injury (output) starting from input variables such as TL indicators. Even though cognitive bias can result from the attribution of “load” as exclusive injury causation, recognizing which indicator is most involved in the onset of injuries would be useful for decision-making in terms of modulating the TL to “reduce the risk of injury”. Decision-making can be considered as a mental simulation that requires the assessment of probability and uncertainty. When a deviation from the probability theory occurs, it is possible to consider a fundamental paradigm known as “heuristics and biases.” [116]

Biases are observed behaviours, while heuristics (a short-cut technique for solving problems that would otherwise be intractable given our cognition processing capacity) are theoretical constructs, including availability [117] and representativeness [117]. *Availability* refers to the tendency to try to estimate the likelihood or frequency of some event by calling to mind examples of the event. The easier it is to recall such examples, the more frequent people judge the event to be, even though it may be biased. For example, the media or some professionals may attribute injuries exclusively to excessive TL. The *representativeness* heuristic is applied when judging the probability of a sample based on a hypothesis related to similarity. For example, people estimate the probability of an event based on how similar it is to a known situation and compare it to a situation, prototype, or stereotype they already have in mind. Cognitive biases, such as the causative explanation of non-contact injuries, can be related to a framework called dual-process or dual-system theories corresponding with the broad distinction between intuitive (System 1) and deliberative (System 2) thinking.

Based on a revised version of this theory carried out by Evans [39], cognitive biases are not exclusive to heuristic processes but can arise from analytic processing as well. However, within the aforementioned theoretical framework, three main principles have been reported. The first one concerns System 2 (analytic), which states that when people think hypothetically, they consider only one possibility or mental model at a time. People focus on only one explicit option when engaged in decision-making [118] and may seldom consider alternatives. This principle could explain the cognitive bias that explains why practitioners often consider one singular factor at a time, such as a TL indicator (e.g. HSR) when trying to understand the cause of an injury.

The second principle asserts that given the current goals of the reasoner, mental models are generated by heuristic or pragmatic processes that are designed to maximize relevance in a particular context. Thus, when people consider a risky prospect, they will normally focus on the most likely outcome first. Similarly, professionals often consider physical exertion (TL) as “the most relevant factor in the field context” as an exclusive cause of injury [119].

The last principle (i.e. the satisficing principle) claims that people’s hypothetical thinking when evaluated by an analytic system is accepted unless there is good reason to reject, modify, or replace it. Of note, cognitive biases arise not only in heuristic systems but also in analytic processing. Stanovich [120] described the tendency to contextualize all problems in light of prior knowledge and beliefs, leading to cognitive biases, such as belief bias.

Accordingly, the Evans framework [114] refers to the cognitive bias underlying the hypothetical reasoning according to which a single factor such as TL can exclusively cause injury. Indeed, the author pointed out that the *fundamental heuristic bias* (in System 1) is essentially the influence of linguistic and attentional factors, as well as contextualization effects. Essentially, people tend to focus selectively on information that is preconsciously cued as “relevant” per the relevance principle. Similarly, a cognitive bias concerns focalism [121], which is the tendency to place too much emphasis on a single factor or piece of information when making judgments or predictions.

The second bias refers to System 2 and is called the *fundamental analytic bias*. This bias involves the tendency to accept a current model (hypothesis, possibility) as the basis of inference, decision, or action without sufficient evaluation or consideration of alternatives (singularity and satisficing principles).

**FIG. 1:**
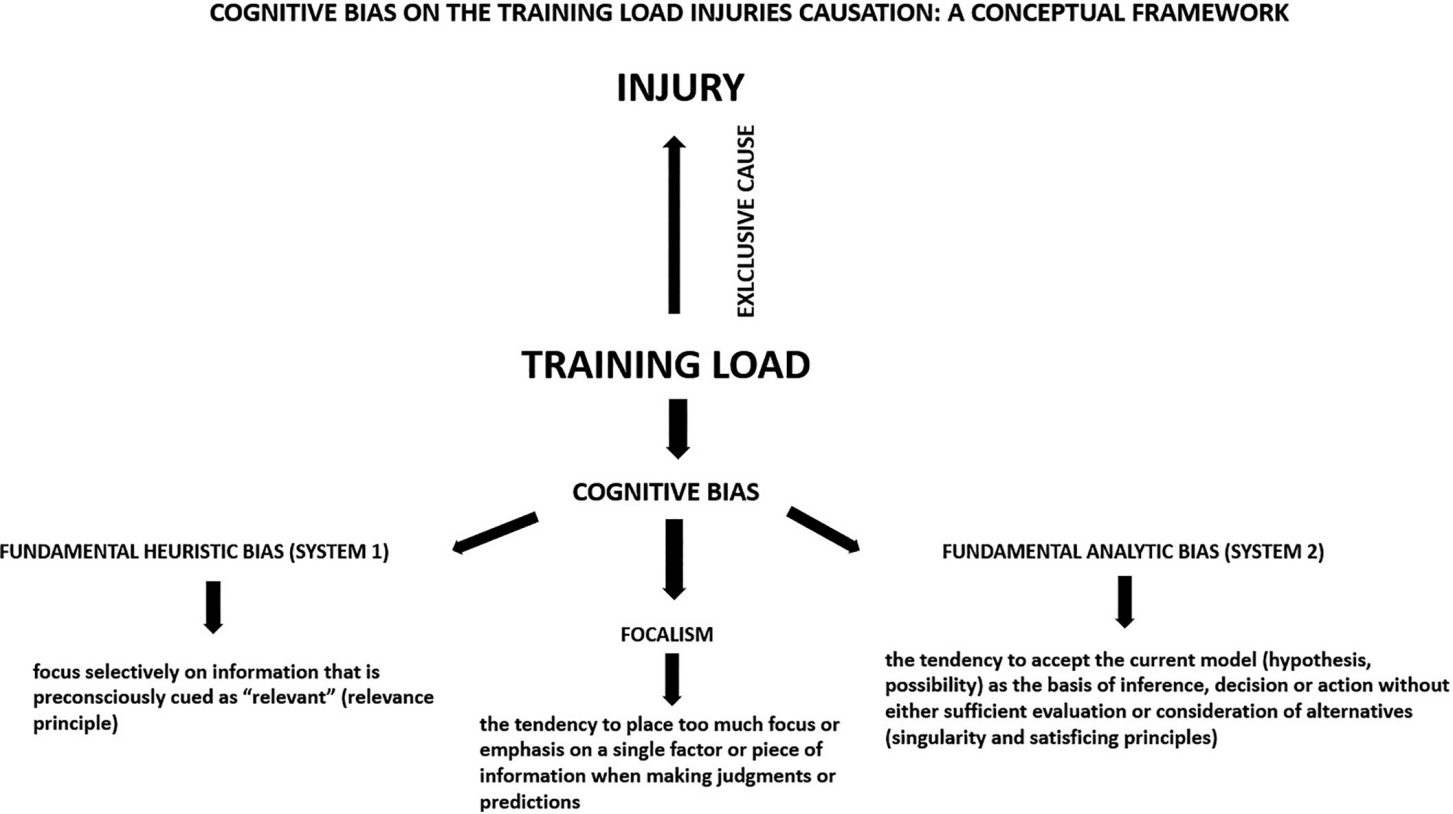
Impact of cognitive bias on training load injury causation: a conceptual framework A conceptual framework related to the impact of cognitive bias on TL-injury causation. Three main cognitive biases (i.e., fundamental heuristic bias, focalism and fundamental analytic bias) are considered when TL is attributed as an exclusive cause of injury.

Based on the previous considerations, it could be stated that when a practitioner, coach, manager, or the media attributes a single factor as the exclusive cause of the onset of non-contact injuries, their reasoning is influenced by cognitive bias. Although it may seem contradictory, stating that TL is not the exclusive cause of an injury does not imply that TL is not strongly implicated in the occurrence of the injury. Indeed, it is logical that injury occurs as a consequence of a certain amount of load where tissue loading exceeds tissue strength [122, 123]. Indeed, Kalkhoven explained the mechanism of athletic injury applying DAGs, identifying two components: the mechanical loading (force) experienced by a tissue and the mechanical strength of that tissue. According to Kalkhoven, one (or both) of these components serves as the conduit through which all causal variables must act, i.e. these two components are reflective of the proximate (ultimate) mechanism of athletic injury [20]. This perspective confirms that TL is not an exclusive cause of injury but is a highly implicated component that interacts with other components (‘internal’ to the athlete, referring to factors related to muscular tissue). Understanding multifactorial and complex phenomena related to human beings’ health requires a high level of knowledge, logical reasoning, evidence-based information, causative inference tools and the elimination of cognitive biases that harm decision-making and the understanding of certain dynamics with related injury reduction strategies. Moreover, qualitative research including a conceptual framework could provide a valuable approach to study the injury aetiology [20]. Figure 1 shows a conceptual framework related to the impact of cognitive bias on TL-injury causation. In the next section we will present an overview of the main conceptual frameworks on sports injuries which attempt to attribute injury to a single cause.

### Conceptual frameworks on sports injuries: is it possible to find an exclusive single “cause” of injury?

The literature reports some frameworks on sports injury to provide useful insights into injury mechanisms and with the attempt to outline appropriate prevention strategies. Kalkhoven’s [9, 20] frameworks have made a significant contribution by incorporating a holistic perspective to understand sports injuries. However, the concept of causeand-effect relationships in injury onset should be approached with caution. To comprehend the cause-and-effect dynamics behind an injury occurrence, practitioners need to address the following questions: Why did a particular athlete sustain an injury at a specific time and under particular circumstances? Is a single factor (such as TL) exclusively responsible for a specific injury?

Building on the information discussed in the previous section, these questions could reveal a cognitive bias, as it is not entirely possible to exclusively attribute a single factor to all occurrences of injuries. Therefore, injuries can be seen as the result of multifactorial human factors. Nonetheless, these authors [9] have conceptualized the complex relationship between relevant potential causal contextual factors, such as physiological and mechanical tissue properties, and the occurrence of injuries, presenting a framework for specific injury mechanisms. Furthermore, the framework introduces the concept of material failure. This concept posits that failure occurs when the strength of a material is surpassed by extreme stress and strain, induced by either the application of a single high-magnitude stress or repeated applications of a load at a certain percentage of the material’s ultimate strength [124].

Based on this concept, most contact and non-contact sports injuries occur as a result of exposure to a singular stress or repetitive stresses and strains [6, 124, 125]. In this context, *stress* refers to the internal forces experienced by a structure (i.e. force per unit of area), while *strain* is defined as the amount of deformation or length change in the direction of an applied force [126].

From a mechanical standpoint, the literature [15] suggests that the primary “cause” of injury is often linked to exceeding the load tolerance or strength of a tissue due to an applied load. While this mechanical perspective has deepened our understanding of injury mechanisms, our viewpoint challenges this “cause-and-effect” relationship, arguing that the assumption of causality is not proven. While this concept outlines a physiological mechanism of injury, injury prevention necessitates an understanding of why a tissue becomes injured (i.e. why it is not adapted to withstand a given load) under specific circumstances at a particular time. Nonetheless, exploring the interactions between structural load tolerance and the application of load provides a valuable contribution to both research and on-field prevention strategies.

According to Kalkhoven’s [9] framework, six causal contextual factors interact with each other, including an individual’s physiological profile and functioning, mechanical properties, and the force applied to the body and the various tissues within. Meanwhile, the load tolerance of the structures subject to injury and the load applied have been related to a deeper level of the framework [9]. The internal stress and strain experienced by specific tissues have also been included as the structural load tolerance and tissue-specific strength. This refers to the main framework’s concept of injury that results if the load applied to a specific structure exceeds the load tolerance.

Based on the perspective provided by the aforementioned framework, the role of tissues (i.e. muscles and tendons) is strongly related to the load experienced by players to implement appropriate injury prevention strategies. In a recent review, Kalkhoven et al. [9] investigated the relationship between TL and injury; they showed how the available TL indicators may relate to the causal pathways of tissue damage and injury incidence. Given the prominent role of mechanical loading (i.e., the forces experienced by specific biological tissues) and a mechanical fatigue failure process that is aetiologically relevant to tissue damage, the authors suggest that the quantification of mechanical loading at the tissue-specific level is crucial to understanding the complex aetiology of injury. Accordingly, TL indicators should quantify or reflect mechanical loading, the mechanical load-response pathway, and, consequently, tissue damage and injuries.

As noted by the authors [9], accurate assessment and quantification of the mechanical load-response require an invasive laboratory setting, making their application unfeasible in real-world scenarios. The external load indicators that best reflect a mechanical load represent a proxy measure of the force applied to muscle tissue and the related structures responsible for high-intensity actions such as HSR, sprint and intense accelerations and decelerations.

While some authors have questioned the validity of GPS-derived metrics as proxies for tissue-level mechanical loads [9, 19], the data-informed approach remains a practical and widely adopted method to support applied physiology and training methodology. Although attributing TL as the exclusive cause of injury reflects a cognitive bias, it is nonetheless highly implicated in injury mechanisms and must be carefully managed [15]. Specifically, controlling key TL components—such as sprint exposure—can guide training prescription, promote positive adaptation, and ultimately contribute to injury prevention.

Nonetheless, this approach represents a possible on-field method for correlating players’ internal responses in order to manage load as a fundamental injury prevention strategy. While mechanical loading and the mechanical load response pathways are crucial for understanding tissue damage, the psychophysiological load-response pathway, including measures of physiological (e.g. heart rate, lactate concentrations) and psychophysiological stress (e.g. ratings of perceived exertion), should also be considered.

However, authors have pointed out that such internal measures poorly correlate with psychophysiological fatigue indicators [127] and do not accurately reflect the mechanical load experienced, leading to inconsistent associations and causations with injury. Although this perspective seems logical and appropriate, monitoring internal load remains a key strategy for understanding an athlete’s response to the external load. This is instrumental in determining whether the training schedule is suitable in a given context and can help avoid suboptimal training practices that could lead to injuries.

Kalkhoven et al. [9] highlighted that linking TL to injury requires considering both mechanical and psychophysiological fatigue. Rather than assuming a direct causal link, injuries should be viewed through a multifactorial lens that accounts for context-specific factors and the complex interaction between external load and tissue response. In this context, a complex systems approach to sports injuries has been advocated by Bittencourt et al. [4], who defined a *complex system* as an entity with parts that interact with each other and which is characterized by inherent non-linearity due to recursive loops and complex interactions among units. Through this approach, practitioners should identify the patterns of relationships among determinants (i.e. specific risk factors related to biomechanics, training characteristics, psychology, and physiology) within a specific context (e.g. soccer or ballet), the regularities (profiles) that concurrently characterize injuries, and the emerging patterns from the complex web of determinants.

According to Philippe and Mansi [128], small changes in a few determinants can lead to large and sometimes unexpected consequences, indicating the non-linear behaviour of determinants interacting with each other. Other authors suggest recognizing the frequent patterns of interaction among multilevel risk factors rather than identifying risk factors at the unit level [129]. For example, Mandarino et al. [130] investigated the interaction between TL markers and both modifiable and non-modifiable risk factors, through a machine learning approach, aiming to predicting non-contact injuries in professional young soccer players [130]. The data-mining technique applied showed that recovery status, internal load markers, and modifiable (height, body mass) and non-modifiable factors (maturity status), interacting each other, can modify the predisposition to risk of injuries [130].

Sports injury research should prioritize the patterns emerging from interactions among multiple determinants, rather than isolated risk factors. For example, ACL injuries in basketball [4] have been linked to factors such as unanticipated events, knee angles, and hip weakness—each influenced by elements such as fatigue, age, sex, and training load, with risk profiles varying according to context (e.g., soccer vs. dance).

No single risk factor can be deemed solely responsible for injury [133]. Instead, identifying risk profiles—patterns of interacting factors leading to injury in the presence of an inciting event—offers a more realistic approach. Machine learning, particularly supervised learning, can help detect such patterns based on player characteristics and risk factor interactions [131, 132]. However, it is important to note that current applications of machine learning in this context have been criticized for a high risk of bias [32].

These frameworks prompt reflection on whether injury causation should be viewed through a single or multilevel lens. In practice, TL is essential for performance but may increase injury risk if not individualized. The following section presents key conceptual frameworks addressing the TL–injury relationship, highlighting limitations, potential sources of bias, and relevant contributions from the literature, despite the lack of causal evidence from RCTs.

### Background: conceptual frameworks on training load and injuries in soccer

Gabbett et al. defined *workloads* as “the cumulative amount of stress placed on an individual from multiple training sessions and games over a period of time” [134]. A combination of intensity, frequency and duration allows one to quantify the demand imposed on a player during both training sessions and matches [135]. Coaches prescribe training in a way that disrupts homeostasis in order to reach optimal adaptations (i.e. the supercompensation cycle). Hence, excessive or insufficient loads should be avoided, as they may lead to detraining, maladaptation, or overtraining among athletes [127, 136, 137].

Accordingly, trying to prescribe training with the optimal load is a difficult challenge for coaches. In light of this, Borresen and Lambert reported that “optimizing training first involves quantifying what the athlete is currently doing” [138]. Usually, this load quantification is permitted to predict the performance, training plan, periodization and monitoring, thus establishing athlete readiness and stress while avoiding injuries [40]. From a logical perspective, injuries are sustained while an athlete performs an activity that determines a workload (both external and internal). Although coaches seek to maximize performance by manipulating the TL, training is necessary to induce positive adaptations by stressing physiological, biomechanical, and metabolic systems. Accordingly, the scientific community should try to understand how the workload is related to injuries and establish whether simply increasing the load increases injury risk. Recently, researchers have provided some practical applications to explain the complex associations between load measures, indicators, indexes, and injuries [6, 31, 34, 38, 134, 139–142]. Notably, while several observational studies have been carried out to understand sport injuries, we highlight that the literature is lacking RCTs—commonly regarded as the ‘gold standard’ for assessing causality [143], as this type of study makes it possible to eliminate the effects of confounding variables [20, 143]. However, conceptual frameworks represent a feasible and appropriate solution to make conceptual distinctions and organise ideas, deconstructing complex phenomena into relevant theories, assumptions, causal links and concepts of interest such as sport injuries [20].

The literature on the complex relationship between load and injuries presents some conceptual frameworks [6, 7, 9, 11, 17, 40, 86, 144]. Windt and Gabbett [11] proposed an updated injury aetiology model, including the effects of workload on injury occurrences. The authors modified previous injury aetiology frameworks [10, 45] by adding the effects of training and competition loads on the dynamic nature of injury risk. The multifactorial nature of injuries is well established in the literature [7].

Meeuwisse proposed a multifactorial injury model highlighting the intrinsic and extrinsic risk factors which predispose athletes to injuries [16]. Some of these intrinsic factors (e.g. age) are non-modifiable, while others (e.g. strength and endurance) are modifiable. Moreover, extrinsic (external) risk factors (e.g. the playing surface, protective equipment, or opponent behaviour) make athletes susceptible to injury. Lastly, injuries occur when a certain inciting event arises wherein the biomechanical stress of the event exceeds the tolerance of the athlete’s tissues [9, 86].

As reported by Windt and Gabbett [11], this multifactorial model has been improved and modified by Bahr et al. [45] and Meeuwisse [10]. The first model provided a more exhaustive description of the biomechanical factors impacting the inciting event. Meanwhile, Meeuwisse proposed a “dynamic, recursive injury aetiology model” highlighting that training/match participation predisposes athletes to injury but also modifies subsequent risk [10] (i.e. it modifies certain risk factors through physiological adaptations to training stress).

However, as reported in the workload-injury aetiology model, workload-injury investigations have focused on the relationship between absolute workloads and injury. Several studies have reported an increase in injury risk with an increase in absolute workload [139]. Nevertheless, an understanding of the role of workload in injury aetiology should be based on the rationale and knowledge of physiology and training methodology while applying appropriate statistical analysis and data interpretation. Furthermore, it would be illogical to think that a low workload may protect athletes from injury risk because the training aims to adapt the athlete to withstand the match demand (i.e. adapt physiological structures to withstand the match demand). In this way, workloads that are too low compared to matchdemand reference values are likely to decrease physical performance and may decrease an athlete’s fitness and preparedness to cope with the match demands. This, in turn, could increase injury risk. Epidemiological studies show that injuries are more likely during matches than training [1, 48, 52, 145].

Another factor relevant to injury risk is how the workload is applied. Studies have examined the relationship between the acute:chronic workload ratio (ACWR), which is calculated based on the acute load (generally one week), chronic load (four weeks), and injury risk [38]. Although Impellizzeri et al. [146] suggested some mathematical and conceptual bias, ACWR has been associated with injury risk [18, 24, 37, 38], but it does not predict injury [30]. Specifically, when ACWR is high (i.e. when spikes in training load occur), an increased risk of injury has been found in rugby players [147] and professional soccer players [18, 24].

Despite the associations found between TL and injuries, the rationale that high workload determines injuries has been refuted by Gabbett, who developed the training-injury prevention paradox model [6]. The author argued that high workloads are essential for developing physical capacity and reducing injury risk, with chronic load acting as a protective factor. To minimize injury risk, weekly load fluctuations should be limited (~10%), and the ACWR should remain within a moderate range to avoid spikes. Despite occasional unavoidable load increases, training should be managed to balance injury prevention with the development of sport-specific physical qualities.

To better understand the complex and multifactorial relationship between TL, injuries, and performance, Gabbett [7] explained why some athletes sustain injuries at an ACWR of ≤ 1.5 while others tolerate extremely high both chronic workloads and ACWR [148–150]. These differences can be explained by the moderators of the workload-injury relationship. Specifically, as explained by Windt et al., “a moderator acts to either increase or decrease injury risk at a given workload” [11, 150]. For example, a spike in workload for athletes with inadequately developed physical capacity (e.g. aerobic fitness, speed, repeated-sprint ability and lower body strength [148, 151]) and low chronic training load [149] have been associated with increased injury risk. The authors suggested that practitioners should stratify players according to age, training and injury history, and physical qualities (moderators of the workload) rather than focusing solely on the ACWR or a single training load indicator. However, the ACWR has been questioned and criticized; accordingly, care should be taken when this metric is associated with injury [9].

Moreover, internal and external load indicators should be considered in combination with well-being and physical readiness data to understand an athlete’s risk of injury [40, 152–154]. In light of these considerations, it is possible to conclude that injuries are not directly and exclusively caused by workloads [8]. Instead, as reported in the updated injury aetiology model developed by Windt and Gabbett [11], “training and competition loads contribute to injury risk through exposing athletes to potentially injurious situations, as well as through their positive and negative effects on numerous modifiable internal risk factors”. This risk modification depends on the total and relative workload applied, leading to positive and negative adaptations. Therefore, workloads can affect injury aetiology through exposure, fitness (i.e. positive adaptation) and fatigue (i.e. negative adaptation).

Researchers have identified the application of workload framework as the primary process by which an athlete is exposed to various external risk factors and potential inciting events [11]. From this perspective, TL is “the vehicle whereby an athlete moves from being a predisposed athlete to a susceptible athlete” [11]. Accordingly, predisposed athletes become susceptible to injury when exposed to a training or competition load. Positive adaptations to modifiable internal risk factors may help explain the training load-injury prevention paradox (i.e. high chronic load and high fitness levels may protect athletes against injuries).

The frameworks mentioned above and the aetiology model [11] allow us to understand the workload-injury relationship more accurately. In summary, when athletes sustain a spike in training that they are unprepared for (i.e. a high ACWR), a larger degree of negative maladaptation will occur, modifying some internal risk factors and increasing their predisposition to sustain an injury in subsequent physical stimuli. Conversely, positive physiological adaptations created by adequate load management (i.e. accumulating high chronic workloads while avoiding training spikes) decrease injury risk.

The misinterpretation of workload-injury investigations may also be derived from statistical analysis, a poor study design, and an improper rationale behind the causality assumption [19]. Given the fundamental role of statistics in the load-injury investigation, researchers should implement a multilevel (mixed) modelling approach. Such an approach is more appropriate for repeated measures (based on the need to quantify workload during a given period) than simple logistic regression models (which assume the same exposure load across individuals).

Multilevel modelling approaches or frailty models [155] can account for correlated outcomes (repeated workload measures among players), including random effects to predict individual athletes’ injury risks. Moreover, specific and more advanced statistical analyses, such as machine learning methods, have been employed for injury prediction. Although no single load factor can be exclusively attributed to injury causation [8], verifying which load indicators are most implicated with injuries can help practitioners implement appropriate prevention strategies. However, several studies have investigated the relationship between workload and injuries in soccer. Jasper [34] reported scarce evidence regarding the relationship between external and internal load and injury or illness. Given the extreme complexity of these factors (i.e. TL and injuries), the literature shows conflicting results. Nevertheless, every athletic injury occurs while athletes are exposed to training and competition workloads regardless of the interaction of risk factors and inciting biomechanical events with a lack of direct causality [8]. Accordingly, despite the lack of direct causality (i.e., TL cannot be considered as the exclusive cause of injury), workload management is one of the main factors that should be considered in injury prevention strategies. Moreover, while practitioners are concerned with workload management (including the monitoring process), an optimal training plan may be essential to increase physical performance and reduce injury risk [8]. In this section we have reported the main frameworks on sports injuries. In the next section we will focus on our new conceptual framework on non-contact injuries and soccer, delving into the possible implications for HSR and sprinting.

#### A new conceptual framework on non-contact injuries in soccer

Drawing on the significant contributions made by previous injury conceptual frameworks, our new framework incorporates several pillars supporting its structure. Specifically, we have elucidated the primary mechanisms underlying hamstring injuries, emphasizing the critical implication of HSR and sprinting on injury occurrence (see Kalkhoven’ framework [20]). While we emphasized the lack of causality when TL is attributed as exclusively “cause” of injury, TL is strongly implicated (i.e., can be considered as one the main factors) in injury occurrence. Accordingly, TL monitoring, focusing on relevant variables such as sprinting through a data-informed approach, represents a necessary and relevant practice to optimize training prescription (aiming to adapt players to withstand match demand) and indirectly reduce injury risk. In shaping our framework’s rationale, we adhered to the multifactorial and complex nature of injuries proposed by Bittencourt et al., according to which the interaction between specific risk factors is linked to injury occurrence. Additionally, we referenced the holistic perspective outlined by Windt and Gabbett [11], which posits that load contributes to injury risk by exposing players to injury situations while providing positive and negative adaptations, ultimately making athletes either stronger (capable of coping with high loads) or weaker (not adapted to cope with high loads).

Through our conceptual perspective, which considers the player’s profile, overall fitness status, and tissue load response based on intrinsic tissue characteristics and strength derived from tissue adaptation, we aim to provide a comprehensive understanding of acute injury events to facilitate the implementation of appropriate prevention strategies. This is achievable through a data-informed approach supported by knowledge of applied physiology and training methodology. It is worth noting that data-informed approach is in contrast to datadriven approaches, which are not supported by any appropriately validated prognostic models for athletic injury risk assessment.

**FIG. 2:**
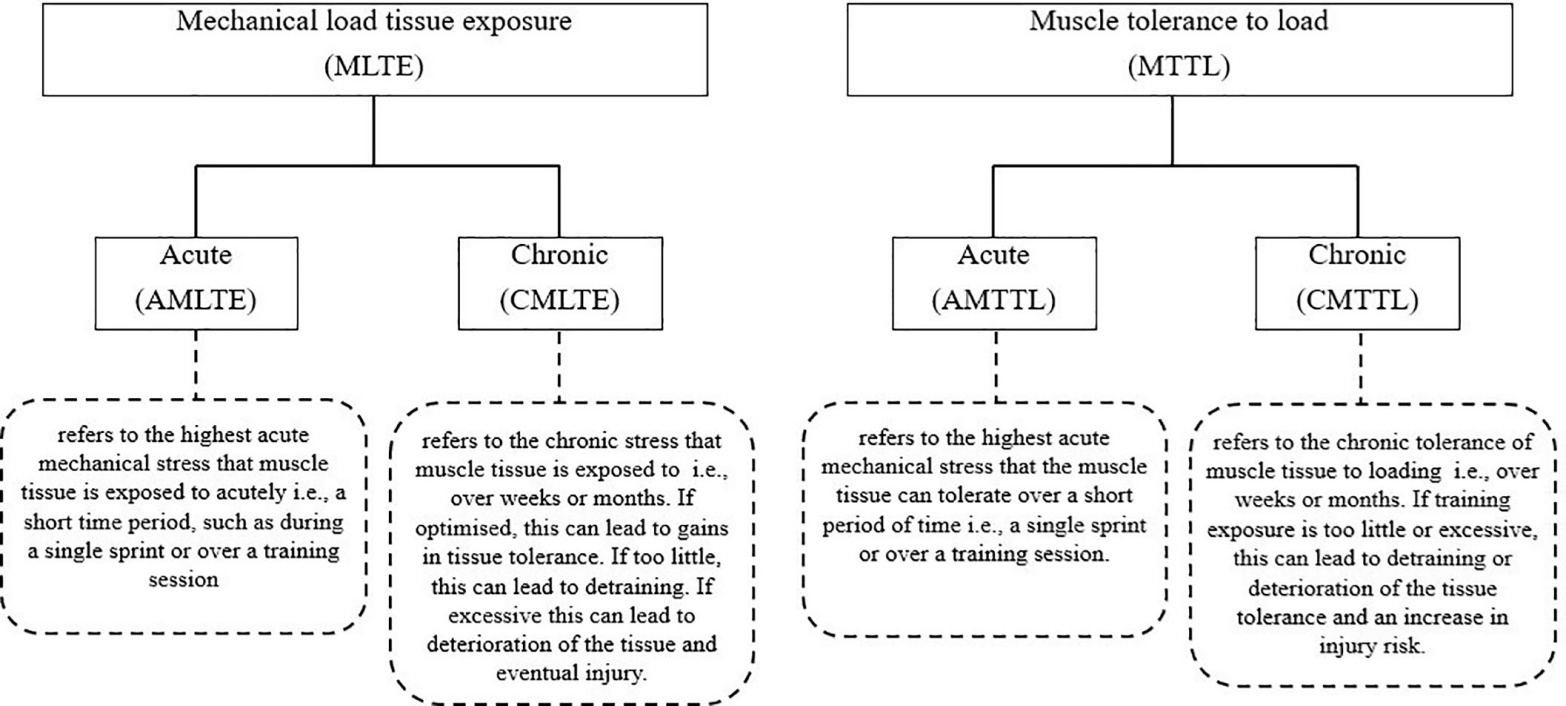
Non-contact injury risk prevention strategies: a conceptual framework As the figure shows, our framework indicates that to mitigate injury risk, practitioners must mainly consider both *acute mechanical load tissue exposure (AMLTE)* and *muscle tolerance to load (MTTL)*. AMLTE represents the highest acute mechanical stress that muscle tissue is exposed to within a specific short period, such as the maximal effort exerted during a sprint, which could lead to a non-contact injury if the tissue has not adapted to such demands. Meanwhile, MTTL indicates the muscle tissue’s ability to tolerate a given load accumulated over time considering both short and long periods. The negative effects of fatigue on injury risk are well documented, and a muscle that is not metabolically adapted is vulnerable to injury.

**FIG. 3:**
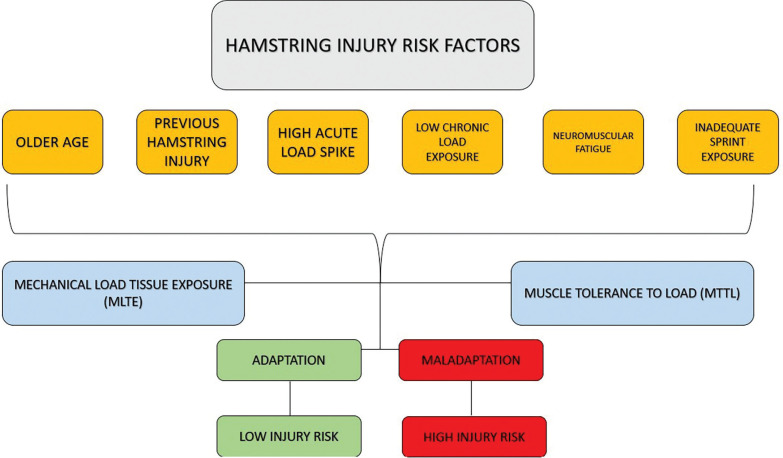
Relationship between hamstring injury risk and AMLTE and MTTL: a conceptual framework Figure 3 shows the conceptual framework illustrating how key risk factors—such as older age, previous hamstring injury, neuromuscular fatigue, low chronic load exposure, and inadequate sprint exposure—simultaneously influence both mechanical load tissue exposure (MLTE) and muscle tolerance to load (MTTL). The quality and balance of these two variables (MLTE and MTTL), rather than the presence of individual risk factors alone, determine whether the athlete undergoes adaptation or maladaptation. Adaptation results in low injury risk, whereas maladaptation increases the likelihood of non-contact hamstring injuries.

Training practices primarily aim to adapt players to withstand match demands while reducing injury occurrence. To achieve optimal physiological and match-related adaptations, we conceptualized that players need to adapt to two main factors related to intensity and volume, as depicted in Figures 2–3. Notably, two relevant training variables such as frequency and density should be considered during the training prescription process. However, we highlighted that our framework has not included frequency and density as both can be considered two complementary variables of volume and intensity. Our framework illustrates that to mitigate injury risk, practitioners must mainly consider both acute mechanical load tissue exposure (AMLTE) and muscle tolerance to load (MTTL). AMLTE represents the highest acute mechanical stress that muscle tissue is exposed to within a specific short period, such as the maximal effort exerted during a sprint, which could lead to a non-contact injury if the tissue has not adapted to such demands. Meanwhile, MTTL indicates the muscle tissue’s ability to tolerate a given load accumulated over time considering both short and long periods. The negative effects of fatigue on injury risk are well documented, and a muscle that is not metabolically adapted is vulnerable to injury.

**FIG. 4:**
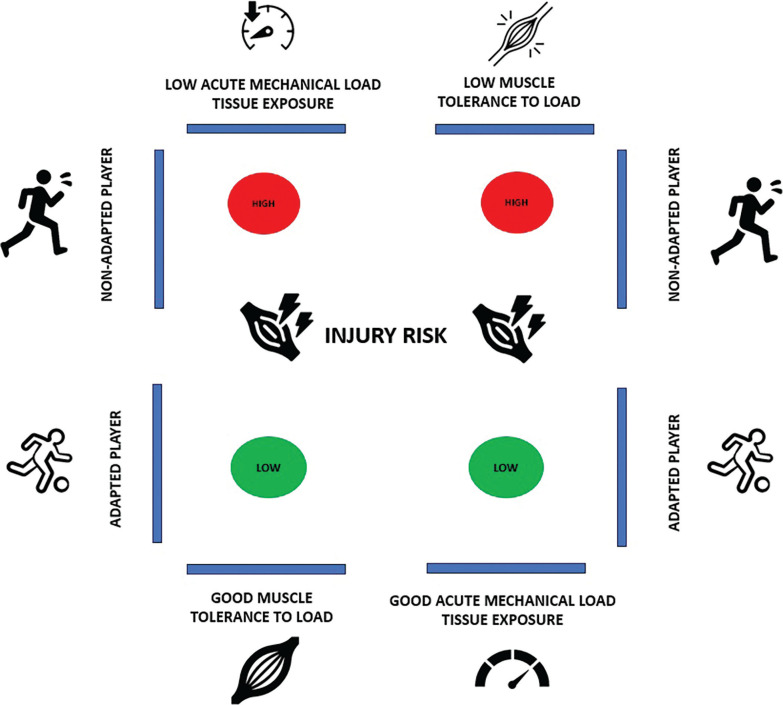
Strong vs weak athlete and non-contact injury risk: a conceptual framework Figure 4 depicts the conceptual relationship between load, player fitness status, and injury risk. When a player is poorly adapted (e.g. if they have a low fitness level or weakness), their muscle load tolerance is low, increasing the risk of injury. Conversely, a strong player (e.g. a player with a high fitness level and good adaptation) has a low risk of injury due to their high load tolerance.

As illustrated in Figure 3, when a player is poorly adapted (e.g. if they have a low fitness level or weakness), their muscle load tolerance is low, increasing the risk of injury. Conversely, a strong player (e.g. a player with a high fitness level and good adaptation) has a low risk of injury due to their high load tolerance. Figure 4 depicts the conceptual relationship between load, player fitness status, and injury risk. Table 1 and Figure 2 illustrate the implications of external load for injury risk in both weak and strong players.

To achieve optimal player adaptation (i.e. the highest fitness level), practitioners must administer appropriate TL while considering both AMLTE and MTTL to protect players from match stimuli and reduce injury and reinjury risk. The conceptual framework outlined above can be better understood by envisioning a real-world scenario that a professional, such as a rehabilitation coach, might encounter. The rehabilitation process aims to adapt players to withstand match demands, such as reaching peak speed and covering HSR distances. By gradually progressing through physiological demands and training methodologies, players reach a level of adaptation that enables them to cope with intense training stimuli. Upon returning to official matches, players’ muscular tissues must be sufficiently adapted to withstand their peak speed and sustain HSR distances based on previous match data. In this process, monitoring biochemical markers [156] (e.g., creatine kinase or inflammatory responses) may offer complementary information on tissue recovery and systemic stress, supporting the early identification of maladaptation and informing decisions related to chronic load tolerance. Therefore, before declaring a player fit for an official match, the rehabilitation coach and medical staff must ensure that the player’s tissues can withstand maximum effort even if only for a short duration.

### How can data mitigate injury occurrence in soccer? A new framework of a data-informed approach and decision-making to reduce injury risk

Load management is widely recognized as one of the primary noncontact injury prevention strategies in soccer [6, 8, 11, 134]. Key factors for effective load management include training methodology, coach experience, and evidence-based knowledge, such as knowledge of sports demands and physiology, which collectively inform appropriate training prescriptions [6, 7, 40, 42]. External and internal load monitoring practices are commonplace in elite soccer and offer invaluable feedback for modifying training regimens and managing external load effectively by employing a data-informed approach. To delve into the relationship between training monitoring and training prescription we refer readers to the review by Pillitteri et al. [92].

**FIG. 5:**
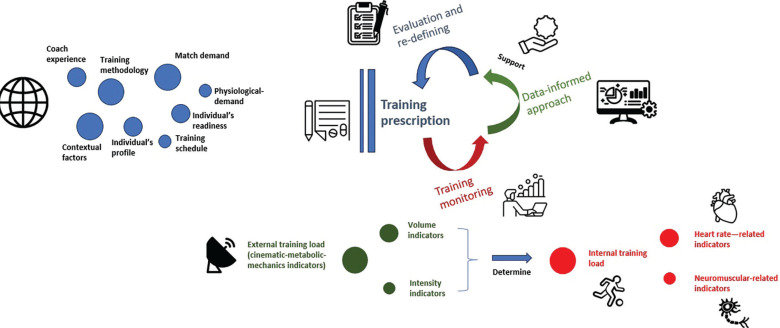
Evidence-based training prescription: a conceptual framework Figures 5 illustrates a data-informed framework depicting the relationship between training prescription and training monitoring prescription in soccer. Specifically, monitoring metabolic, HSR, and neuromuscular external load indicators (comprising both volume and intensity) provides practitioners with essential information on soccer physical performance, representing key factors that should be considered in training prescriptions. This allows the management of training prescriptions to be altered or adjusted based on collected data.

**FIG. 6:**
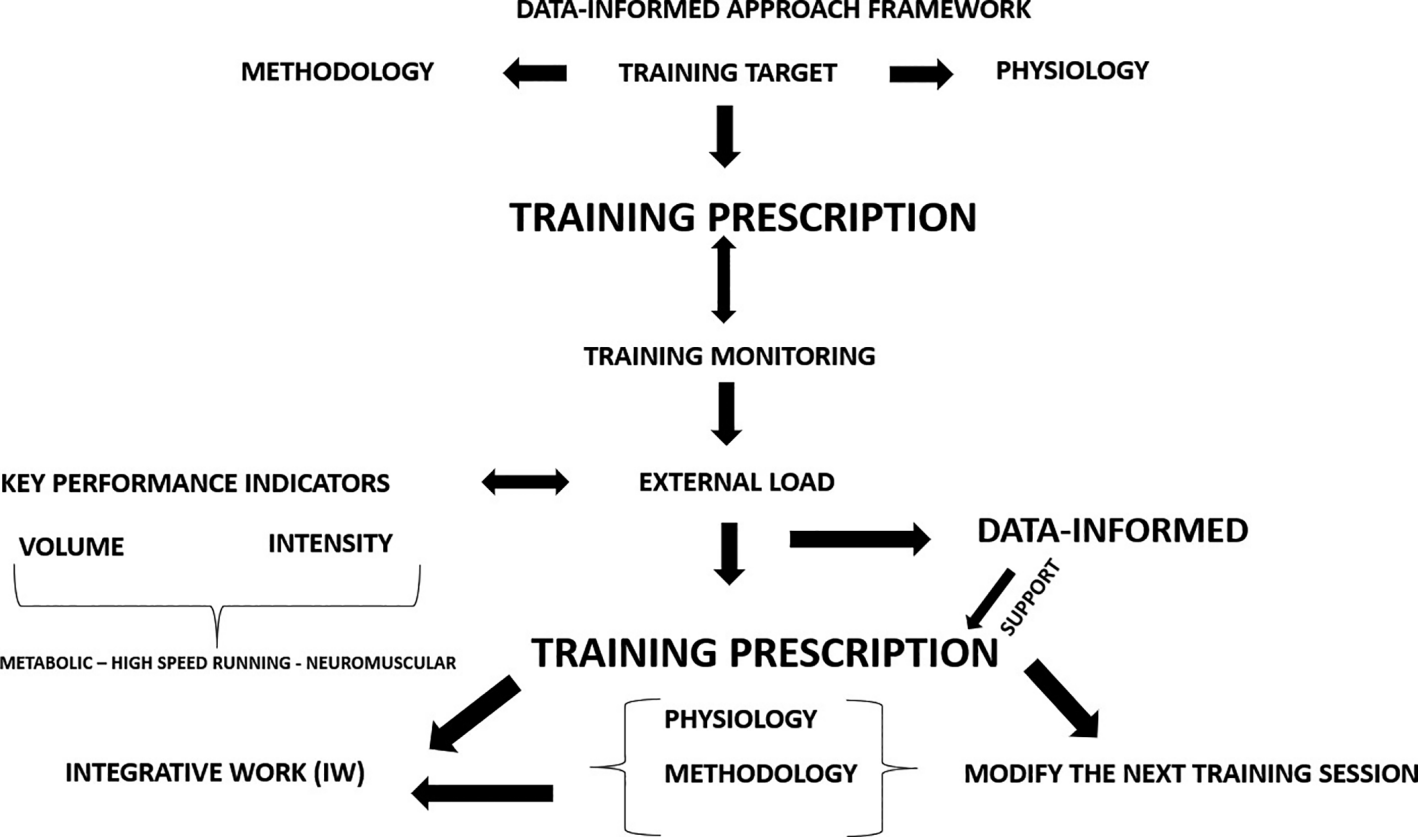
Decision-making based on a data-informed approach: a conceptual framework The training prescription is supported by a data-informed approach. In fact, both external and internal load data provide information to make an appropriate decision on the training prescription. In particular, it may be possible to increase the load through integrative work (IW) at the end of the section, as well as to modify the next training section by increasing or decreasing the load (considering the individualization process in which the load is managed for each player).

**FIG. 7:**
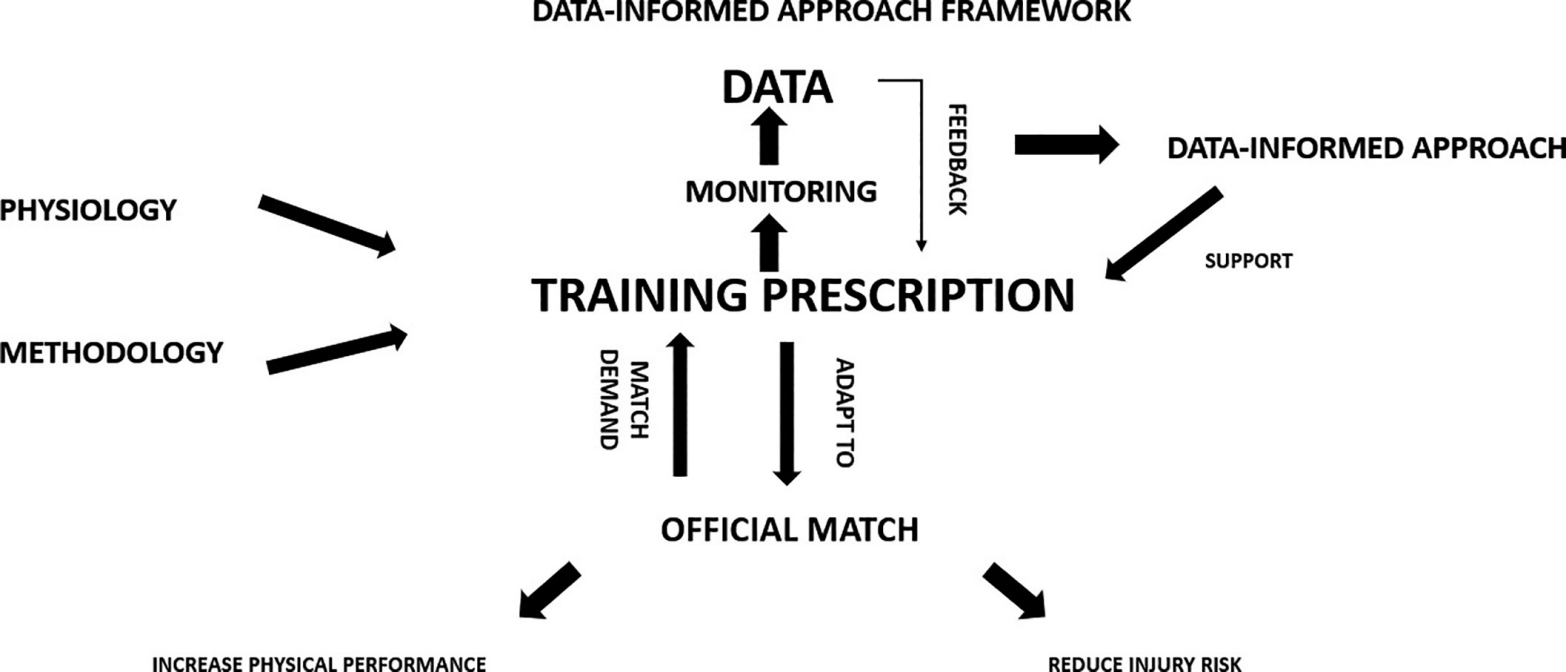
Data-informed approach to training prescription: a conceptual framework Figure 7 shows the implications of a data-informed approach for training prescription. Training prescription should be mainly based on training methodology (including training principles), knowledge of physiology and the information derived from match demand (e.g., GPS data). Data support training prescription providing useful feedback to make a decision on training load management.

Figures 5–7 illustrate a data-informed framework depicting the relationship between training prescription and training monitoring prescription in soccer. Specifically, monitoring metabolic, HSR, and neuromuscular external load indicators (comprising both volume and intensity) provides practitioners with essential information on soccer physical performance, representing key factors that should be considered in training prescriptions. This allows the management of training prescriptions to be altered or adjusted based on collected data. For example, integrative work may be implemented after training sessions to achieve training goals, and subsequent training days’ prescriptions can be adjusted as necessary. Managing training load in soccer requires a strategic approach to ensure that both starters and non-starters receive appropriate stimuli to sustain physical readiness and reduce injury risk [157]. A critical challenge in this context is the reduced exposure to HSR and sprinting (i.e., > 25.2 km/h) typically experienced by non-starters due to limited match participation. Integrating sprint-specific work for these athletes is further complicated by microcycle constraints and the need to periodize TL without compromising recovery or readiness.

To address this imbalance, well-structured compensatory training becomes essential. These sessions should target the replication of match demands through tailored sprint exposures and peak speed development. Effective strategies may include a combination of sprint training and soccer-specific drills, each serving complementary purposes. In detail, sprint training (without ball) aims to elicit neuromuscular adaptations—particularly in the hamstrings—by improving acceleration mechanics and enhancing maximal sprinting capacity. This should consist of linear sprints of at least 40–50 m performed at maximal effort, with adequate recovery intervals to ensure optimal muscle recruitment and to avoid fatigue-induced technical decline. Soccer-specific drills (with ball) should be designed to replicate HSR and sprint demands observed in competition. Riboli et al. [158–160] recommend organizing drills with a relative area of approximately 220 m^2^ per player to optimize sprint exposure while preserving tactical and technical fidelity. Such drills should incorporate large playing spaces, dynamic transitions, and rule constraints that promote match-like sprinting actions.

This integrative compensatory approach not only contributes to bridging the gap in physical stimulus for non-starters but also supports chronic load development (CMTTL), neuromuscular adaptation, and the prevention of detraining effects, ultimately reducing injury susceptibility.

Tables 1–2 provide an example of how a data-informed approach can be applied in a real-world soccer scenario. The data-informed approach promotes optimal load management, as it enables modifications to training prescriptions based on feedback derived from monitoring data. Our framework emphasizes the supportive role of data-informed approaches in training prescription. However, relying exclusively on a data-informed approach for training prescription may introduce errors or cognitive biases, such as focalism, as discussed in previous sections [39, 114, 121].

**TABLE 1:** Relationship between High-Speed running/Peak Speed, and Injury Risk considering the differences between strong and weak players: an example from the microcycle

	PLAYER A STRONG PLAYER												

	1° MICROCYCLE						2° MICROCYCLE					

	MD-4	MD-3	MD-2	MD-1	MD	MD+1/-6	MD+2/-5	MD+3/-4	MD+4/-3	MD+5/-2	MD+6/-1	MD
DISTANCE > 25.2 km/h (m)	40	190	120	30	**210**	DAY OFF	40	90	200	100	20	**250**
	
PEAK SPEED (km/h)	26	33	26	29	**33**		26	28	35	30	29	**34**

**PLAYER B WEAK PLAYER**												

	**1° MICROCYCLE**						**2° MICROCYCLE**					

	**MD-4**	**MD-3**	**MD-2**	**MD-1**	**MD**	**MD+1/-6**	**MD+2/-5**	**MD+3/-4**	**MD+4/-3**	**MD+5/-2**	**MD+6/-1**	**MD**

DISTANCE > 25.2 km/h (m)	0	30	0	0	**70**	DAY OFF	0	20	40	10	0	**200**
	
PEAK SPEED (km/h)	23	27	26	27	**29.5**		26	25	28	27	28	**31**


**PLAYER A**							**PLAYER B**					

Strong player: adapted to cope with acute and chronic external load							Weak player: not adapted to cope with acute and chronic external load					
**2° MICROCYCLE**							**2° MICROCYCLE**					
Match Day (MD)							Match Day (MD)					
Distance covered at high-speed running (> 25.2 km/h): 250 m Peak speed reached: 34 km/h (95% of individual max speed) INTERNAL LOAD RPE: 3							Distance covered at high-speed running (> 25.2 km/h): 200 m Peak speed reached: 31 km/h (95% of individual max speed) INTERNAL LOAD RPE: 5					
**ATHLETE FITNESS STATUS: EL/IL 250/3 = 83.3**							**ATHLETE FITNESS STATUS: EL/IL 200/5 = 40**					
**TRAINING LOAD 2° MICROCYCLE**							**TRAINING LOAD** **2° MICROCYCLE**					
**EXTERNAL TRAINING LOAD VOLUME**							**EXTERNAL TRAINING LOAD VOLUME**					
Distance covered at high-speed running (> 25.2 km/h): 450 m							Distance covered at high-speed running (> 25.2 km/h): 70 m					
**EXTERNAL TRAINING LOAD INTENSITY**							**EXTERNAL TRAINING LOAD INTENSITY**					
Peak speed reached: 35 km/h							Peak speed reached: 28 km/h					
TRAINING/MATCH LOAD RATIO: 450/250 = 1.8*							**TRAINING/MATCH LOAD RATIO: 70/200 = 0.35**					

**OUTCOME:** Player A has been gradually and well adapted to cope with this high external load which carries a low injury risk during both MD and next training session. Specifically, player A has been prepared to withstand match demand as he travelled 1.8 the amount of distance > 25.2 km/h compared to match. Accordingly, the physiological structures involved to perform can support the high stress related to match demand. Moreover, player A present a higher fitness status than player B based on the EL/ IL ratio.							**OUTCOME:** Player B shows a spike for high-speed running and consequently high injury risk during both MD and next training session. Specifically, player B has not been prepared to withstand match demand as he travelled 0.3 the amount of distance > 25.2 km/h compared to match. Accordingly, the physiological structures involved to perform cannot support the high stress related to match demand. Moreover, player B present a lower fitness status than player A based on the EL/ IL ratio.					
*PREPARED TO WITHSTAND MATCH DEMAND BASED ON DISTANCE > 25.2 km/h (m)							*NOT PREPARED TO WITHSTAND MATCH DEMAND BASED ON DISTANCE > 25.2 km/h (m)					

**TABLE 2:** A data-informed approach based on the external load data (sprinting) collected in the microcycle considering playing position

MD+3/-3 Aim: development of Sprinting ability Target fullback: 180 m (distance > 25.2 km/h)	

PLAYER A	PLAYER B
Playing position: fullback Reference value (official match): Distance covered in Sprinting (> 25.2 km/h): 200 m Peak speed reached: 34 km/h	Playing position: fullback Reference value (official match): Distance covered in Sprinting (> 25.2 km/h): 200 m Peak speed reached: 34 km/h
**EXTERNAL TRAINING LOAD VOLUME**	**EXTERNAL TRAINING LOAD VOLUME**
Distance covered in Sprinting (> 25.2 km/h): 90 m Peak speed reached: 27.2 km/h (80% peak speed)	Distance covered in Sprinting (> 25.2 km/h): 250 m Peak speed reached: 32.3 km/h (80% peak speed)
DECISION MAKING BASED ON DATA INFORMED: - INTEGRATIVE WORK AT THE END OF THE TRAINING 3 × 30 m SPRINT 3 × 40 m SPRINT	DECISION MAKING BASED ON DATA INFORMED: - NO INTEGRATIVE WORK - MANAGE LOAD (VOLUME) IN THE NEXT TRAINING SESSION

**OUTCOME:** Based on the reference value (physical demand derived from official match), player A shows low distance covered in Sprinting (> 25.2 km/h). Accordingly, additional integrative work (sprint) will be administered at the end of the training session.	**OUTCOME:** Based on the reference value (physical demand derived from official match), player A shows excessive distance covered in Sprinting (> 25.2 km/h). Accordingly, the load will be managed in the next training session.

Sound training practice should begin with an understanding of applied physiology and training methodologies, and data should be collected during monitoring to provide feedback for effective training management. Through the feedback provided by data, it is possible to profile drills, players, and match days (within the microcycle) and to understand the physical demands of soccer (including monitoring official matches). However, data-informed programming can be susceptible to conceptual errors if the process is driven solely by numbers, which should be viewed as supportive rather than prescriptive. In this regard, it is essential to consider additional data sources— such as recovery status, wellness indicators (e.g., sleep quality, muscle soreness, and fatigue), readiness to train, and nutritional and hydration metrics—which represent critical components in evidencebased decision-making processes for training prescription and player management, although they fall outside the primary scope of the present work. Additionally, the structure and sequencing of sprintintensive sessions within the weekly microcycle are fundamental in regulating both mechanical load tissue exposure (MLTE) and MTTL. Recent findings by Buchheit et al. [93] emphasize that incorporating sprint exposures > 95% of maximal sprint speed on MD-2 can contribute to reducing injury risk, provided that prior training days (e.g., MD-3 and MD-4) are managed to minimize residual fatigue. Mismanagement of microcycle sequencing—such as poor distribution of high-speed efforts or insufficient progression throughout the week—may result in excessive acute tissue stress without developing adequate chronic tolerance, thereby increasing the likelihood of maladaptation and non-contact injury. A well-designed weekly structure with progressive load exposure is thus essential to harmonize both acute and chronic MLTE and CMTTL, aiming to optimize both performance while reducing injury risk. For a more detailed understanding of microcycle planning and the integration of sprint and HSR strategies, readers are encouraged to consult the work of Buchheit et al. [93], which offers valuable insights into how structured weekly periodization can contribute to injury mitigation in elite soccer.

## CONCLUSIONS

Injuries, particularly hamstring injuries, represent a high-cost burden for elite soccer clubs.

With the aim of translating research into practical insights for practitioners, we have summarized the main findings on non-contact injury in soccer, and described the different conceptual frameworks of soccer injuries. Finally, we present a new conceptual framework on non-contact soccer injuries focusing on the contribution offered by data-informed approaches to implement non-contact injury prevention strategies.

Hamstring injuries have been linked to HSR and sprinting activities, primarily due to the high eccentric loading experienced during the late swing phase and early stance phase. However, the relationship between TL and injury occurrence is complex, and attributing a cause-effect relationship between a single factor and injury may be prone to cognitive biases.

Predicting, associating, or establishing a statistical relationship between a risk factor and injury occurrence lacks the explanatory power necessary for causal inferences, and therefore fails to explain why an athlete was injured at a particular time and under specific circumstances. Nonetheless, recognizing potential injury risk factors such as HSR can help practitioners exercise caution in real-world scenarios, such as HSR-related training sessions.

We have developed a conceptual framework that highlights two main factors related to intensity and volume: acute mechanical load tissue exposure (AMLTE) and muscle tolerance to load (MTTL). We have done this to mitigate the risk of injury and reinjury, drawing from previous contributions of conceptual frameworks, qualitative research, and field experience. By focusing on both AMLTE and MTTL, practitioners can move towards optimal “protective” player adaptation (i.e. improving the athlete’s fitness level to withstand match demands) and reduce injury risk.

In a real-world context, coupling optimal training practices with appropriate TL management should be regarded as the best prevention strategy to mitigate injury risk. Assessing an athlete’s fitness status (i.e. whether their tissues are adapted to withstand external stimuli) to administer a significant load, such as HSR, may facilitate effective injury prevention strategies, considering that daily invasive assessments of physiological properties are not feasible. Consequently, employing an indirect method, such as a data-informed approach alongside sound training methodology (while also considering applied physiology), promotes the understanding of whether an athlete can tolerate the EL that induces an appropriate internal response (i.e. load tolerance).

Data-informed approaches should be viewed as supportive tools for training prescription, as they help achieve optimal adaptation and increase a player’s physical status, thereby potentially reducing injury risk. By exclusively focusing on data while neglecting both applied physiology in sports and training methodology, practitioners may commit reasoning errors (cognitive biases), which may not foster injury prevention. Indeed, due to the complex nature of non-contact hamstring injuries, along with the absence of an appropriately validated prognostic model for predicting injury risk, practitioners should be aware of their underlying biases. This is particularly important to consider in cases where selected interventions restrict athletes (e.g., training, or competitive exposure), especially when such interventions are imposed on athletes against their will, and without robust athletic injury predictions as a justification.

The cause-effect relationship regarding injury needs to be implemented thought accurate scientific methods such as RCTs or causative inference tools such as frameworks, model, or DAGs. In the future, it may be useful to optimize machine learning approaches methodology to explore the interactions between various risk factors and intrinsic athlete-related aspects that may concurrently contribute to acute injury events.

## Data Availability

All data supporting the findings of this review are available in this published article or as a supplement.

## Summary and applied recommendations

– Epidemiological studies indicate high hamstring injury rates, particularly during matches and among professional male players. The focus on hamstring injuries centres on their biomechanical causes during high-speed running (HSR) and sprinting, especially during the late swing and early stance/initial contact phases, where the muscle’s role as an eccentric brake is crucial in decelerating knee extension.
– – Despite hypothesised pathways through which TL can influence injury risk, TL cannot be considered the exclusive cause of injury. Injury is a complicated phenomenon, and understanding the multifactorial and complex nature of non-contact injuries requires a high level of knowledge, logical reasoning, evidence-based information, the elimination of cognitive biases and the understanding of certain dynamics with related injury reduction strategies.
– Our framework illustrates that to mitigate injury risk, practitioners must mainly consider both acute mechanical load tissue exposure (AMLTE) and muscle tolerance to load (MTTL). AMLTE represents the highest acute mechanical stress that muscle tissue is exposed to within a specific short period, such as the maximal effort exerted during a sprint, which could lead to a non-contact injury if the tissue has not adapted to such demands. Meanwhile, MTTL indicates the muscle tissue’s ability to tolerate a given load accumulated over time considering both short and long periods. The negative effects of fatigue on injury risk are well documented, and a muscle that is not metabolically adapted is vulnerable to injury.
– Potentially due to the complex nature of hamstring functioning during dynamic sporting activities, existing measures of hamstring functioning and tolerance are limited and offer minimal utility for the prediction of hamstring injuries. Accordingly, for managing hamstring injury risk, a data-informed approach is advocated. This approach involves carefully integrating available data into the overall decision-making process regarding training load management by considering the unique situation of each athlete (e.g., training needs, professional goals, event schedule), reflecting a more balanced, individualised, and holistic approach to managing hamstring injury risk. This is in contrast to more rigid data-driven approaches which may unjustifiably restrict an athlete’s training against their will, and which are not supported by any appropriately validated prognostic models for athletic injury risk assessment.
– Data-informed approaches should be regarded as practical and appropriate tools for guiding training prescriptions, offering insights to support various athlete-focused goals. However, due to the current limitations in accurately assessing injury risk using existing metrics, training load measures should be applied cautiously, particularly when restricting training outputs. Such decisions should be made collaboratively with the athlete and the organization, while also considering broader sporting objectives, such as performance and development goals. Solely attributing athletic injury to training load data risks oversimplifying the issue and ignoring the complex interplay of causal factors that contribute to injury outcomes. This reductionist approach can lead to reasoning errors and cognitive biases, ultimately causing tension between relevant stakeholders when training load is restricted unjustifiably.

## References

[1] Ekstrand J, Hägglund M, Waldén M. Injury incidence and injury patterns in professional football: the UEFA injury study. Br J Sports Med. 2011; 45(7):553–558. doi: 10.1136/bjsm.2009.060582. 19553225

[2] Ekstrand J, Waldén M, Hägglund M. Hamstring injuries have increased by 4% annually in men’s professional football, since 2001: a 13-year longitudinal analysis of the UEFA Elite Club injury study. Br J Sports Med. 2016; 50(12):731–737. doi: 10.1136/bjsports-2015-095359. 26746908

[3] Vermeulen R, van Dyk N, Whiteley R, et al. Injury-inciting circumstances of sudden-onset hamstring injuries: video analyses of 63 match injuries in male professional football players in the Qatar Stars League (2013–2020). Br J Sports Med. 2024; 58(20):1196–1204. doi: 10.1136/bjsports-2023-106722. 39242176

[4] Bittencourt NFN, Meeuwisse WH, Mendonça LD, Nettel-Aguirre A, Ocarino JM, Fonseca ST. Complex systems approach for sports injuries: moving from risk factor identification to injury pattern recognition—narrative review and new concept. Br J Sports Med. 2016; 50(21):1309–1314. doi: 10.1136/bjsports-2015-095850. 27445362

[5] Buchheit M, Settembre M, Hader K, McHugh D. From high-speed running to hobbling on crutches: A machine learning perspective on the relationships between training doses and match injury trends. Sport Perform Sci Rep. 2023; 216:1–11.

[6] Gabbett TJ. The training-injury prevention paradox: should athletes be training smarter and harder? Br J Sports Med. 2016; 50(5):273–280. doi: 10.1136/bjsports-2015-095788. 26758673 PMC4789704

[7] Gabbett TJ. Debunking the myths about training load, injury and performance: empirical evidence, hot topics and recommendations for practitioners. Br J Sports Med. 2020; 54(1):58–66. doi: 10.1136/bjsports-2018-099784. 30366966

[8] Impellizzeri FM, Menaspà P, Coutts AJ, Kalkhoven J, Menaspà MJ. Training load and its role in injury prevention, part I: back to the future. J Athl Train. 2020; 55(9):885–892. doi: 10.4085/1062-6050-500-19. 32991701 PMC7534945

[9] Kalkhoven JT, Watsford ML, Coutts AJ, Edwards WB, Impellizzeri FM. Training load and injury: causal pathways and future directions. Sports Med. 2021; 51:1137–1150. doi: 10.1007/s40279-020-01413-6. 33400216

[10] Meeuwisse WH, Tyreman H, Hagel B, Emery C. A dynamic model of etiology in sport injury: the recursive nature of risk and causation. Clin J Sports Med. 2007; 17(3):215–219. doi: 10.1097/JSM.0b013e3180592a48. 17513916

[11] Windt J, Gabbett TJ. How do training and competition workloads relate to injury? The workload—injury aetiology model. Br J Sports Med. 2017; 51(5):428–435. doi: 10.1136/bjsports-2016-096040. 27418321

[12] Gregson W, Di Salvo V, Varley MC, et al. Harmful association of sprinting with muscle injury occurrence in professional soccer match-play: a two-season, league-wide exploratory investigation from the Qatar Stars League. J Sci Med Sport. 2020; 23(2):134–138. doi: 10.1016/j.jsams.2019.08.289. 31591064

[13] Bache-Mathiesen LK, Andersen TE, Dalen-Lorentsen T, et al. A new statistical approach to training load and injury risk: separating the acute from the chronic load. Biol Sport. 2024; 41(1):119–134. doi: 10.5114/biolsport.2024.127388. PMC10765439 38188114

[14] Pillitteri G, Petrigna L, Ficarra S, et al. Relationship between external and internal load indicators and injury using machine learning in professional soccer: a systematic review and meta-analysis. Res Sports Med. 2024; 1–37. doi: 10.1080/15438627.2023.2297190. 38146925

[15] Kalkhoven JT, Lukauskis-Carvajal M, Sides DL, McLean BD, Watsford ML. A conceptual exploration of hamstring muscle–tendon functioning during the late-swing phase of sprinting: the importance of evidence-based hamstring training frameworks. Sports Med. 2023; 53(12):2321–2346. doi: 10.1007/s40279-023-01904-2. 37668895 PMC10687166

[16] Meeuwisse W. Assessing causation in sport injury: a multifactorial model. Clin J Sports Med. 1994; 166–170. doi: 10.1097/00042752-199407000-00004.

[17] Impellizzeri FM, Marcora SM, Coutts AJ. Internal and external training load: 15 years on. Int J Sports Physiol Perform. 2019; 14(2):270–273. doi: 10.1123/ijspp.2018-0935. 30614348

[18] Andrade R, Wik EH, Rebelo-Marques A, et al. Is the acute: chronic workload ratio (ACWR) associated with risk of time-loss injury in professional team sports? A systematic review of methodology, variables and injury risk in practical situations. Br J Sports Med. 2020; 50(9):1613–1635. doi: 10.1007/s40279-020-01308-6. 32572824

[19] Impellizzeri FM, McCall A, Ward P, Bornn L, Coutts AJ. Training load and its role in injury prevention, part 2: conceptual and methodologic pitfalls. J Athl Train. 2020; 55(9):893–901. doi: 10.4085/1062-6050-501-19. 32991699 PMC7534938

[20] Kalkhoven JT. Athletic injury research: frameworks, models and the need for causal knowledge. Sports Med. 2024; 54(5):1121–1137. doi: 10.1007/s40279-024-02008-1. 38507193 PMC11127898

[21] Buchheit M, Settembre M, Hader K, McHugh D. From high-speed running to hobbling on crutches: A machine learning perspective on the relationships between training doses and match injury trends. Sport Perform Sci Rep. 2023; 216:1–11.

[22] Buchheit M, Settembre M, Hader K, McHugh D. Exposures to near-tomaximal speed running bouts during different turnarounds in elite football: association with match hamstring injuries. Biol Sport. 2023; 40(4):1057–1067. doi: 10.5114/biolsport.2023.125595. 37867737 PMC10588569

[23] Edouard P, Mendiguchia J, Guex K, et al. Sprinting: a potential vaccine for hamstring injury? Sport Perform Sci Rep. 2019.

[24] Bowen L, Gross AS, Gimpel M, Bruce-Low S, Li FX. Spikes in acute: chronic workload ratio (ACWR) associated with a 5–7 times greater injury rate in English Premier League football players: a comprehensive 3-year study. Br J Sports Med. 2020; 54(12):731–738. doi: 10.1136/bjsports-2018-099422. 30792258 PMC7285788

[25] Windt J, Zumbo BD, Sporer B, MacDonald K, Gabbett TJ. Why do workload spikes cause injuries, and which athletes are at higher risk? Mediators and moderators in workload–injury investigations. Br J Sports Med. 2017; 51(5):993–994. doi: 10.1136/bjsports-2016-097255. 28274916

[26] McArdle W, Katch F, Katch V. Exercise physiology: nutrition, energy, and human performance. 2010: Lippincott Williams & Wilkins.

[27] Foster C. Monitoring training in athletes with reference to overtraining syndrome. Med Sci Sports Exerc. 1998; 30(7):1164–1168. doi: 10.1097/00005768-199807000-00023. 9662690

[28] Hostrup M, Bangsbo J. Performance adaptations to intensified training in top-level football. Sports Med. 2023; 53(3):577–594. doi: 10.1007/s40279-022-01791-z. 36380164 PMC9667002

[29] Rossi A, Pappalardo L, Cintia P. A narrative review for a machine learning application in sports: an example based on injury forecasting in soccer. Sports. 2021; 10(1):5. doi: 10.3390/sports10010005. 35050970 PMC8822889

[30] Rossi A, Pappalardo L, Cintia P, Iaia FM, Fernàndez J, Medina D. Effective injury forecasting in soccer with GPS training data and machine learning. PLoS One. 2018; 13(7):e0201264. doi: 10.1371/journal.pone.0201264. 30044858 PMC6059460

[31] Vallance E, Sutton-Charani N, Imoussaten A, Montmain J, Perrey S. Combining internal and external training loads to predict non-contact injuries in soccer. Appl Sci. 2020; 10(15):5261. doi: 10.3390/app10155261.

[32] Bullock GS, Mylott J, Hughes T, Nicholson KF, Riley RD, Collins GS. Just how confident can we be in predicting sports injuries? A systematic review of the methodological conduct and performance of existing musculoskeletal injury prediction models in sport. Sports Med. 2022; 52(10):2469–2482. doi: 10.1007/s40279-022-01698-9. 35689749

[33] Coyne JOC, Gregory Haff G, Coutts AJ, Newton RU, Nimphius S. The current state of subjective training load monitoring—a practical perspective and call to action. Sports Med Open. 2018; 4:1–10. doi: 10.1186/s40798-018-0172-x. 30570718 PMC6301906

[34] Jaspers A, Brink MS, Probst SG, Frencken WG, Helsen WF. Relationships between training load indicators and training outcomes in professional soccer. J Sports Med Sci. 2017; 47(3):533–544. doi: 10.1007/s40279-016-0591-0. 27459866

[35] Hulin BT, Gabbett TJ, Blanch P, Chapman P, Bailey D, Orchard JW. Spikes in acute workload are associated with increased injury risk in elite cricket fast bowlers. Br J Sports Med. 2014; 48(8):708–712. doi: 10.1136/bjsports-2013-092524. 23962877

[36] Duhig S, Shield AJ, Opar D, Gabbett TJ, Ferguson C, Williams M. Effect of high-speed running on hamstring strain injury risk. Br J Sports Med. 2016; 50(24):1536–1540. doi: 10.1136/bjsports-2015-095679. 27288515

[37] Dalen-Lorentsen T, Bjørneboe J, Clarsen B, Vagle M, Fagerland MW, Andersen TE. Does load management using the acute: chronic workload ratio prevent health problems? A cluster randomized trial of 482 elite youth footballers of both sexes. Br J Sports Med. 2021; 55(2):108–114. doi: 10.1136/bjsports-2020-103003. 33036995

[38] Griffin A, Kenny IC, Comyns TM, Lyons M. The association between the acute: chronic workload ratio and injury and its application in team sports: a systematic review. Sports Med. 2020; 50(3):561–580. doi: 10.1007/s40279-019-01218-2. 31691167

[39] Evans J. Hypothetical thinking: Dual processes in reasoning and judgment. 2007: Psychology Press. doi: 10.4324/9780367823832.

[40] Gabbett TJ, Nassis GP, Oetter E, et al. The athlete monitoring cycle: a practical guide to interpreting and applying training monitoring data. Br J Sports Med. 2017; 51(20):1451–1452. doi: 10.1136/bjsports-2016-097298. 28646100

[41] Dhahbi W, Materne O, Chamari K. Rethinking knee injury prevention strategies: joint-by-joint training approach paradigm versus traditional focused knee strengthening. Biol Sport. 2025; 42(4):59–65. doi: 10.5114/biolsport.2025.148544. PMC12490306 41048234

[42] Buchheit M, Laursen PB. High-intensity interval training, solutions to the programming puzzle: Part I: cardiopulmonary emphasis. Sports Med. 2013; 43(5):313–338. doi: 10.1007/s40279-013-0029-x. 23539308

[43] Stølen T, Chamari K, Castagna C, Wisløff U. Physiology of soccer: an update. Sports Med. 2005; 35:501–536. doi: 10.2165/00007256-200535060-00004. 15974635

[44] Ekblom B. Applied physiology of soccer. Sports Med. 1986; 3:50–60. doi: 10.2165/00007256-198603010-00005. 3633120

[45] Bahr R, Krosshaug T. Understanding injury mechanisms: a key component of preventing injuries in sport. Br J Sports Med. 2005; 39(6):324–329. doi: 10.1136/bjsm.2005.018341. 15911600 PMC1725226

[46] Fuller CW, Ekstrand J, Junge A, et al. Consensus statement on injury definitions and data collection procedures in studies of football (soccer) injuries. J Sports Sci. 2006; 16(2):83–92. doi: 10.1136/bjsm.2005.025270. 16533346

[47] Waldén M, Mountjoy M, McCall A, et al. Football-specific extension of the IOC consensus statement: methods for recording and reporting of epidemiological data on injury and illness in sport 2020. Br J Sports Med. 2023; 57(21):1341–1350. doi: 10.1136/bjsports-2022-106405. 36609352 PMC10646851

[48] Ekstrand J, Waldén M, Hägglund M. Hamstring injuries have increased by 4% annually in men’s professional football, since 2001: a 13-year longitudinal analysis of the UEFA Elite Club injury study. Br J Sports Med. 2016; 50(12):731–737. doi: 10.1136/bjsports-2015-095359. 26746908

[49] Ekstrand J. Keeping your top players on the pitch: the key to football medicine at a professional level. 2013, BMJ Publishing Group Ltd and British Association of Sport and Exercise Medicine. doi: 10.1136/bjsports-2013-092771.

[50] Pulici L, Certa D, Zago M, Volpi P, Esposito F. Injury burden in professional European football (soccer): systematic review, meta-analysis, and economic considerations. Clin J Sport Med. 2023; 33(4):450–457. doi: 10.1097/JSM.0000000000001107. 36730365

[51] Eckard TG, Padua DA, Hearn DW, Pexa BS, Frank BS. The relationship between training load and injury in athletes: a systematic review. Sports Med. 2018; 48(8):1929–1961. doi: 10.1007/s40279-018-0951-z. 29943231

[52] Ekstrand J, Hägglund M, Waldén M. Epidemiology of muscle injuries in professional football (soccer). Am J Sports Med. 2011; 39(6):1226–1232. doi: 10.1177/0363546510395879. 21335353

[53] Faude O, Rößler R, Junge A. Football injuries in children and adolescent players: are there clues for prevention? Sports Med. 2013; 43(9):819–837. doi: 10.1007/s40279-013-0061-x. 23723046

[54] Hägglund M, Waldén M, Magnusson H, Kristenson K, Bengtsson H, Ekstrand J. Injuries affect team performance negatively in professional football: an 11-year follow-up of the UEFA Champions League injury study. Br J Sports Med. 2013; 47(12):738–742. doi: 10.1136/bjsports-2013-092215. 23645832

[55] López-Valenciano A, Ruiz-Pérez I, Garcia-Gómez A, et al. Epidemiology of injuries in professional football: a systematic review and meta-analysis. Br J Sports Med. 2020; 54(12):711–718. doi: 10.1136/bjsports-2018-099577. 31171515 PMC9929604

[56] Szymski D, Krutsch V, Achenbach L, et al. Epidemiological analysis of injury occurrence and current prevention strategies on international amateur football level during the UEFA Regions Cup 2019. BMC Musculoskelet Disord. 2022; 142(2):271–280. doi: 10.1007/s00402-021-03861-9. PMC8783909 33740068

[57] Hägglund M, Waldén M, Ekstrand J. Injury incidence and distribution in elite football—a prospective study of the Danish and the Swedish top divisions. Br J Sports Med. 2005; 15(1):21–28. doi: 10.1111/j.1600-0838.2004.00395.x. 15679568

[58] Chamari K, Rekik RN, Chaabane M, et al. Evolution of injury burden in Qatari professional football – 8 season data from the Aspetar Injury and Illness Surveillance Programme. Biol Sport. 2025; 42(1):201–209. doi: 10.5114/biolsport.2025.139089. 39758174 PMC11694202

[59] Waldén M, Hägglund M, Werner J, Ekstrand J. The epidemiology of anterior cruciate ligament injury in football (soccer): a review of the literature from a gender-related perspective. Knee Surg Sports Traumatol Arthrosc. 2011; 19:3–10. doi: 10.1007/s00167-010-1172-7. 20532868

[60] Pulici L, Certa D, Zago M, Volpi P, Esposito F. Injury burden in professional European football (soccer): systematic review, meta-analysis, and economic considerations. 2022; p. 10.1097. doi: 10.1097/JSM.0000000000001107. 36730365

[61] Croisier JL, Ganteaume S, Binet J, Genty M, Ferret JM. Strength imbalances and prevention of hamstring injury in professional soccer players: a prospective study. Am J Sports Med. 2008; 36(8):1469–1475. doi: 10.1177/0363546508316764. 18448578

[62] Engebretsen AH, Myklebust G, Holme I, Engebretsen L, Bahr R. Intrinsic risk factors for hamstring injuries among male soccer players: a prospective cohort study. Am J Sports Med. 2010; 38(6):1147–1153. doi: 10.1177/0363546509358381. 20335507

[63] Hägglund M, Waldén M, Ekstrand J. Previous injury as a risk factor for injury in elite football: a prospective study over two consecutive seasons. Br J Sports Med. 2006; 40(9):767–772. doi: 10.1136/bjsm.2006.026609. 16855067 PMC2564391

[64] Hägglund M, Waldén M, Ekstrand J. Risk factors for lower extremity muscle injury in professional soccer: the UEFA Injury Study. Am J Sports Med. 2013; 41(2):327–335. doi: 10.1177/0363546512470634. 23263293

[65] Askling C, Karlsson J, Thorstensson A. Thorstensson. Hamstring injury occurrence in elite soccer players after preseason strength training with eccentric overload. Scand J Med Sci Sports. 2003; 13(4):244–250. doi: 10.1034/j.1600-0838.2003.00312.x. 12859607

[66] Askling C, Saartok T, Thorstensson A. Type of acute hamstring strain affects flexibility, strength, and time to return to pre-injury level. Br J Sports Med. 2006; 40(1):40–44. doi: 10.1136/bjsm.2005.018879. 16371489 PMC2491922

[67] Askling CM, Malliaropoulos N, Karlsson J. High-speed running type or stretching-type of hamstring injuries makes a difference to treatment and prognosis. Br J Sports Med. 2012; 46(2):86–87. doi: 10.1136/bjsports-2011-090534. 22171341

[68] Petersen J, Thorborg K, Nielsen MB, Budtz-Jørgensen E, Hölmich P. Preventive effect of eccentric training on acute hamstring injuries in men’s soccer: a cluster-randomized controlled trial. Am J Sports Med. 2011; 39(11):2296–2303. doi: 10.1177/0363546511419277. 21825112

[69] Chebbi S, Chamari K, Van Dyk N, Gabbett T, Tabben M. Hamstring Injury Prevention for Elite Soccer Players: A Real-World Prevention Program Showing the Effect of Players’ Compliance on the Outcome. J Strength Cond Res. 2022; 36(5):1383–1388. doi: 10.1519/JSC.0000000000003505. 33590986

[70] Green B, Bourne MN, van Dyk N, Pizzari T. Recalibrating the risk of hamstring strain injury (HSI): A 2020 systematic review and meta-analysis of risk factors for index and recurrent hamstring strain injury in sport. Br J Sports Med. 2020; 54(18):1081–1088. doi: 10.1136/bjsports-2019-100983. 32299793

[71] Kenneally-Dabrowski CJB, Brown NAT, Lai AKM, Perriman D, Spratford W, Serpell BG. Late swing or early stance? A narrative review of hamstring injury mechanisms during high-speed running. Scand J Med Sci Sports. 2019; 29(8):1083–1091. doi: 10.1111/sms.13437. 31033024

[72] Garrett WE Jr. Muscle strain injuries. Am J Sports Med. 1996; 24(6_ suppl):S2–S8. 8947416

[73] Lieber RL, Fridén J. Muscle damage is not a function of muscle force but active muscle strain. J Appl Physiol. 1993; 74(2):520–526. doi: 10.1152/jappl.1993.74.2.520. 8458765

[74] Mann R, Sprague P. A kinetic analysis of the ground leg during sprint running. Res Q Exerc Sport. 1980; 51(2):334–348. doi: 10.1080/02701367.1980.10605202. 7394297

[75] Heiderscheit BC, Hoerth DM, Chumanov ES, Swanson SC, Thelen BJ, Thelen DG. Identifying the time of occurrence of a hamstring strain injury during treadmill running: a case study. Clin Biomech. 2005; 20(10):1072–1078. doi: 10.1016/j.clinbiomech.2005.07.005. 16137810

[76] Schache AG, Kim HJ, Morgan DL, Pandy MG. Hamstring muscle forces prior to and immediately following an acute sprinting-related muscle strain injury. Gait Posture. 2010; 32(1):136–140. doi: 10.1016/j.gaitpost.2010.03.006. 20395142

[77] Askling CM, Tengvar M, Saartok T, Thorstensson A. Acute first-time hamstring strains during high-speed running: a longitudinal study including clinical and magnetic resonance imaging findings. Am J Sports Med. 2007; 35(2):197–206. doi: 10.1177/0363546506294679. 17170160

[78] Orchard JW. Hamstrings are most susceptible to injury during the early stance phase of sprinting. 2012, BMJ Publishing Group Ltd and British Association of Sport and Exercise Medicine; p. 88–89. doi: 10.1136/bjsports-2011-090127. 21930513

[79] Sun Y, Wei S, Zhong Y, Fu W, Li L, Liu Y. How joint torques affect hamstring injury risk in sprinting swing–stance transition. Med Sci Sports Exerc. 2015; 47(2):373. doi: 10.1249/MSS.0000000000000404. 24911288 PMC4323551

[80] Thelen DG, Chumanov ES, Best TM, Swanson SC, Heiderscheit BC. Simulation of biceps femoris musculotendon mechanics during the swing phase of sprinting. Med Sci Sports Exerc. 2005; 37(11):1931–1938. doi: 10.1249/01.mss.0000176674.42929.de. 16286864

[81] Askling CM, Tengvar M, Saartok T, Thorstensson A. Acute first-time hamstring strains during slow-speed stretching: clinical, magnetic resonance imaging, and recovery characteristics. Am J Sports Med. 2007; 35(10):1716–1724. doi: 10.1177/0363546507303563. 17567821

[82] Jones RI, Ryan B, Todd AI. Muscle fatigue induced by a soccer match-play simulation in amateur Black South African players. J Sports Sci. 2015; 33(12):1305–1311. doi: 10.1080/02640414.2015.1022572. 25764064

[83] Schwiete C, Roth C, Skutschik C, et al. Effects of muscle fatigue on exerciseinduced hamstring muscle damage: a three-armed randomized controlled trial. Eur J Appl Physiol. 2023; p. 1–17. doi: 10.1007/s00421-023-05234-z. PMC10616225 37330434

[84] Schuermans J, Van Tiggelen D, Danneels L, Witvrouw E. Susceptibility to hamstring injuries in soccer: a prospective study using muscle functional magnetic resonance imaging. Am J Sports Med. 2016; 44(5):1276–1285. doi: 10.1177/0363546515626538. 26912281

[85] Bramah C, Mendiguchia J, Dos’Santos T, Morin JB. Exploring the role of sprint biomechanics in hamstring strain injuries: a current opinion on existing concepts and evidence. Sports Med. 2023; p. 1–11. doi: 10.1007/s40279-023-01925-x. PMC11052868 37725240

[86] Aiello F, Di Claudio C, Fanchini M, et al. Do non-contact injuries occur during high-speed running in elite football? Preliminary results from a novel GPS and video-based method. J Sci Med Sport. 2023; 26(9):465–470. doi: 10.1016/j.jsams.2023.07.007. 37544819

[87] Teixeira JE, Forte P, Ferraz R, et al. Monitoring accumulated training and match load in football: A systematic review. Int J Environ Res Public Health. 2021; 18(8):3906. doi: 10.3390/ijerph18083906. 33917802 PMC8068156

[88] Danielsson A, Horvath A, Senorski C, et al. The mechanism of hamstring injuries–a systematic review. BMC Musculoskelet Disord. 2020; 21:1–21. doi: 10.1186/s12891-020-03658-8. PMC7526261 32993700

[89] Gualtieri A, Rampinini E, Dello Iacono A, Beato M. High-speed running and sprinting in professional adult soccer: current thresholds definition, match demands and training strategies. A systematic review. Front Sports Active Living. 2023; 5:1116293. doi: 10.3389/fspor.2023.1116293. PMC9968809 36860737

[90] Clemente FM, Rabbani A, Conte D, et al. Training/match external load ratios in professional soccer players: A full-season study. Int J Environ Res Public Health. 2019; 16(17):3057. doi: 10.3390/ijerph16173057. 31443592 PMC6747517

[91] Baptista I, Johansen D, Seabra A, Pettersen SA. Position specific player load during match-play in a professional football club. PLoS One. 2018; 13(5):e0198115. doi: 10.1371/journal.pone.0198115. 29795703 PMC5967838

[92] Pillitteri G, Clemente FM, Sarmento H, et al. Translating player monitoring into training prescriptions: Real world soccer scenario and practical proposals. Int J Sports Sci Coaching. 2024; p. 17479541241289080. doi: 10.1177/17479541241289080.

[93] Buchheit M, Douchet T, Settembre M, et al. The 11 Evidence-Informed and Inferred Principles of Microcycle Periodization in Elite Football. Sport Perform Sci Rep. 2024; 218.

[94] Buchheit M, Sandua M, Berndsen J, et al. Loading patterns and programming practices in elite football: insights from 100 elite practitioners. 2021; 153:v1.

[95] Buchheit M, Settembre M, Hader K, McHugh D. Planning the microcycle in elite football: to rest or not to rest? Injuries and days off-feet in elite football. doi: 10.1123/ijspp.2022-0146. 36596310

[96] Beato M, Drust B, Iacono AD. Implementing high-speed running and sprinting training in professional soccer. Int J Sports Med. 2021; 42(04):295–299. doi: 10.1055/a-1302-7968. 33291180

[97] Liu Y, Sun Y, Zhu W, Yu J. The late swing and early stance of sprinting are most hazardous for hamstring injuries. J Sport Health Sci. 2017; 6(2):133. doi: 10.1016/j.jshs.2017.01.011. 30356597 PMC6188991

[98] Van Hooren B, Bosch F. Is there really an eccentric action of the hamstrings during the swing phase of high-speed running? Part I: A critical review of the literature. J Sports Sci. 2017; 35(23):2313–2321. doi: 10.1080/02640414.2016.1266018. 27937671

[99] Van Hooren B, Bosch F. Bosch. Is there really an eccentric action of the hamstrings during the swing phase of high-speed running? Part II: Implications for exercise. J Sports Sci. 2017; 35(23):2322–2333. doi: 10.1080/02640414.2016.1266018. 27935419

[100] Biewener A. Muscle function in vivo: a comparison of muscles used for elastic energy savings versus muscles used to generate mechanical power. Am Zoologist. 1998; 38(4):703–717. doi: 10.1093/icb/38.4.703.

[101] McBride JM. Muscle actuators, not springs, drive maximal effort human locomotor performance. J Sports Sci Med. 2021; 20(4):766. doi: 10.52082/jssm.2021.766. 35321123 PMC8488820

[102] Holt NC, Roberts TJ, Askew GN. The energetic benefits of tendon springs in running: is the reduction of muscle work important? J Exp Biol. 2014; 217(24):4365–4371. doi: 10.1242/jeb.112813. 25394624 PMC4375839

[103] Lindstedt SL, LaStayo PC, Reich TE. When active muscles lengthen: properties and consequences of eccentric contractions. Physiology. 2001; 16(6):256–261. doi: 10.1152/physiologyonline.2001.16.6.256. 11719600

[104] Chumanov ES, Heiderscheit BC, Thelen DG. The effect of speed and influence of individual muscles on hamstring mechanics during the swing phase of sprinting. J Biomech. 2007; 40(16):3555–3562. doi: 10.1016/j.jbiomech.2007.05.026. 17659291

[105] Van Hooren B, Bosch F. Influence of muscle slack on high-intensity sport performance: A review. Strength Cond J. 2016; 38(5):75–87. doi: 10.1519/SSC.0000000000000251.

[106] Jönhagen S, Ericson MO, Németh G, Eriksson E. Amplitude and timing of electromyographic activity during sprinting. Scand J Med Sci Sports. 1996; 6(1):15–21. doi: 10.1111/j.1600-0838.1996.tb00064.x. 8680937

[107] van Dyk N, Behan FP, Whiteley R. Including the Nordic hamstring exercise in injury prevention programmes halves the rate of hamstring injuries: a systematic review and meta-analysis of 8459 athletes. Br J Sports Med. 2019; 53(21):1362–1370. doi: 10.1136/bjsports-2018-100045. 30808663

[108] Rice N, Bemis CM, Daley MA, Nishikawa K. Understanding Muscle Function during in vivo Locomotion Using a Novel Muscle Avatar Approach. 2020, Northern Arizona University. doi: 10.1242/jeb.244721. 37334740

[109] Brenner B, Eisenberg E. Muscle mechanics and biochemical kinetics. Molecular mechanisms in muscular contraction. 1990:77–149. doi: 10.1007/978-3-662-11289-2_1.

[110] Prince C, Morin JB, Mendiguchia J, et al. Sprint specificity of isolated hamstring-strengthening exercises in terms of muscle activity and force production. Front Sports Active Living. 2021; 2:609636. doi: 10.3389/fspor.2020.609636. PMC7859261 33554110

[111] Lolli L, Bahr R, Weston M, et al. No association between perceived exertion and session duration with hamstring injury occurrence in professional football. Scand J Med Sci Sports. 2020; 30(3):523–530. doi: 10.1111/sms.13591. 31663176

[112] Whiteley R, Gregson W, Bahr R, et al. High-speed running during match-play before and after return from hamstring injury in professional footballers. Scand J Med Sci Sports. 2022; 32(10):1502–1509. doi: 10.1111/sms.14219. 35934809

[113] Smith SC Jr. Multiple risk factors for cardiovascular disease and diabetes mellitus. Am J Med. 2007; 120(3):S3–S11. doi: 10.1016/j.amjmed.2007.01.002. 17320520

[114] Evans J. Bias in human reasoning: Causes and consequences. 1989: Psychology Press.

[115] Tabben M, Verhagen E, Warsen M, et al. Obstacles and opportunities for injury prevention in professional football in Qatar: exploring the implementation reality. BMJ Open Sport Exerc Med. 2023; 9(1):e001370. doi: 10.1136/bmjsem-2022-001370. PMC10008252 36919121

[116] Tversky A, Kahneman D. Kahneman. Judgment under Uncertainty: Heuristics and Biases: Biases in judgments reveal some heuristics of thinking under uncertainty. Science. 1974; 185(4157):1124–1131. doi: 10.1126/science.185.4157.1124. 17835457

[117] Pohl R. Cognitive illusions: A handbook on fallacies and biases in thinking, judgment and memory. 2004: Psychology Press.

[118] Legrenzi P, Girotto V, Johnson-Laird PN. Johnson-Laird. Focusing in reasoning and decision making. Cognition. 1993; 49(1–2):37–66. doi: 10.1016/0010-0277(93)90035-t. 8287674

[119] Weston M. Training load monitoring in elite English soccer: a comparison of practices and perceptions between coaches and practitioners. Sci Med Football. 2018; 2(3):216–224. doi: 10.1080/24733938.2018.1427883.

[120] Stanovich K. Who is rational?: Studies of individual differences in reasoning. 1999: Psychology Press.

[121] Ehrlinger J, Readinger W, Kim B. Decision-making and cognitive biases. Encyclopedia of Mental Health. 2016; 12(3):83–7. doi: 10.1016/B978-0-12-397045-9.00206-8.

[122] Edwards WB. Modeling overuse injuries in sport as a mechanical fatigue phenomenon. Exerc Sport Sci Rev. 2018; 46(4):224–231. doi: 10.1249/JES.0000000000000163. 30001271

[123] Kalkhoven JT, Watsford ML, Impellizzeri FM. A conceptual model and detailed framework for stressrelated, strain-related, and overuse athletic injury. J Sci Med Sport. 2020; 23(8):726–734. doi: 10.1016/j.jsams.2020.02.002. 32111566

[124] Gallagher S, Heberger JR. Examining the interaction of force and repetition on musculoskeletal disorder risk: a systematic literature review. Hum Factors. 2013; 55(1):108–124. doi: 10.1177/0018720812449648. 23516797 PMC4495348

[125] Fung YC. Biomechanics: mechanical properties of living tissues. 2013: Springer Science & Business Media.

[126] Hart NH, Nimphius S, Rantalainen T, Ireland A, Siafarikas A, Newton RU. Mechanical basis of bone strength: influence of bone material, bone structure and muscle action. J Musculoskelet Neuronal Interact. 2017; 17(3):114. 28860414 PMC5601257

[127] Halson SL. Monitoring training load to understand fatigue in athletes. Sports Med. 2014; 44 Suppl 2(Suppl 2):S139–47. doi: 10.1007/s40279-014-0253-z. 25200666 PMC4213373

[128] Philippe P, Mansi O. Nonlinearity in the epidemiology of complex health and disease processes. Theor Med Bioethics. 1998; 19:591–607. doi: 10.1023/a:1009979306346. 10051792

[129] Hulme A, Finch CF. Finch. From monocausality to systems thinking: a complementary and alternative conceptual approach for better understanding the development and prevention of sports injury. Injury Epidemiol. 2015; 2(1):1–12. doi: 10.1186/s40621-015-0064-1. PMC4673096 26691678

[130] Mandorino M, Figueiredo A, Cima G, Tessitore. A data mining approach to predict non-contact injuries in young soccer players. Int J Comput Sci Sport. 2021; 20(2):147–163. 10.2478/ijcss-2021-0009.

[131] Rossi A, Pappalardo L, Cintia P, Iaia FM, Fernàndez J, Medina D. Effective injury forecasting in soccer with GPS training data and machine learning. PLoS One. 2018; 13(7):e0201264. doi: 10.1371/journal.pone.0201264. 30044858 PMC6059460

[132] Vallance E, Sutton-Charani N, Imoussaten A, Montmain J, Perrey S. Combining internal-and externaltraining-loads to predict non-contact injuries in soccer. Appl Sci. 2020; 10(15):5261. doi: 10.3390/app10155261.

[133] Bahr R. Why screening tests to predict injury do not work—and probably never will…: a critical review. Br J Sports Med. 2016; 50(13):776–80. doi: 10.1136/bjsports-2016-096256. 27095747

[134] Gabbett TJ, Whyte DG, Hartwig TB, Wescombe H, Naughton GA. The relationship between workloads, physical performance, injury and illness in adolescent male football players. J Sci Med Sport. 2014; 44(7):989–1003. doi: 10.1007/s40279-014-0179-5. 24715614

[135] Smith DJ. A framework for understanding the training process leading to elite performance. J Sci Med Sport. 2003; 33(15):1103–1126. doi: 10.2165/00007256-200333150-00003. 14719980

[136] Halson SL, Jeukendrup AE. Does overtraining exist? J Sports Med. 2004; 34(14):967–981. doi: 10.2165/00007256-200434140-00003. 15571428

[137] Issurin VB. Benefits and limitations of block periodized training approaches to athletes’ preparation: A review. Sports Med. 2016; 46(3):329–38. doi: 10.1007/s40279-015-0425-5. 26573916

[138] Borresen J, Lambert MI. The quantification of training load, the training response and the effect on performance. J Sports Med. 2009; 39(9):779–795. doi: 10.2165/11317780-000000000-00000. 19691366

[139] Drew MK, Finch CF. The relationship between training load and injury, illness and soreness: a systematic and literature review. J Sports Med. 2016; 46(6):861–883. doi: 10.1007/s40279-015-0459-8. 26822969

[140] Montini M, Rocchi JE. Monitoring Training Load in Soccer: The ROMEI J Strength Cond Res. 2022; 36(9):2566–2572. doi: 10.1519/JSC.0000000000003875. 33136773

[141] Verstappen S, van Rijn RM, Cost R, Stubbe JH. The association between training load and injury risk in elite youth soccer players: a systematic review and best evidence synthesis. Sports Med Open. 2021; 7(1):6. doi: 10.1186/s40798-020-00296-1. 33428001 PMC7801562

[142] Chamari K, Bahr R. Training for elite sport performance: Injury risk management also matters! Int J Sports Physiol Perform. 2016; 11(5):561–2. doi: 10.1123/IJSPP.2016-0207. 27464009

[143] Bothwell LE, Greene JA, Podolsky SH, Jones DS. Assessing the gold standard—lessons from the history of RCTs. N Engl J Med. 2016; 374(22):2175–2181. doi: 10.1056/NEJMms1604593. 27248626

[144] Jeffries AC, Marcora SM, Coutts AJ, Wallace L, McCall A, Impellizzeri FM. Development of a revised conceptual framework of physical training for use in research and practice. Sports Med. 2021:1–16. doi: 10.1007/s40279-021-01551-5. 34519982

[145] Pfirrmann D, Herbst M, Ingelfinger P, Simon P, Tug S. Analysis of injury incidences in male professional adult and elite youth soccer players: a systematic review. J Sci Med Sport. 2016; 51(5):410–424. doi: 10.4085/1062-6050-51.6.03. PMC5013706 27244125

[146] Impellizzeri FM, Tenan MS, Kempton T, Novak A, Coutts AJ. Acute: chronic workload ratio: conceptual issues and fundamental pitfalls. Int J Sports Physiol Perform. 2020; 15(6):907–913. doi: 10.1123/ijspp.2019-0864. 32502973

[147] Hulin BT, Gabbett TJ, Lawson DW, Caputi P, Sampson JA. The acute: chronic workload ratio predicts injury: high chronic workload may decrease injury risk in elite rugby league players. Br J Sports Med. 2016; 50(4):231–236. doi: 10.1136/bjsports-2015-094817. 26511006

[148] Malone S, Owen A, Mendes B, Hughes B, Collins K, Gabbett TJ. High-speed running and sprinting as an injury risk factor in soccer: Can well-developed physical qualities reduce the risk? J Sci Med Sport. 2018; 21(3):257–262. doi: 10.1016/j.jsams.2017.05.016. 28595870

[149] Malone S, Roe M, Doran DA, Gabbett TJ, Collins KD. Protection against spikes in workload with aerobic fitness and playing experience: the role of the acute: chronic workload ratio on injury risk in elite Gaelic football. Int J Sports Physiol Perform. 2017; 12(3):393–401. doi: 10.1123/ijspp.2016-0090. 27400233

[150] Windt J, Zumbo BD, Sporer B, MacDonald K, Gabbett TJ. Why do workload spikes cause injuries, and which athletes are at higher risk? Mediators and moderators in workload–injury investigations. 2017, BMJ Publishing Group Ltd and British Association of Sport and Exercise Medicine; p. 993–994. doi: 10.1136/bjsports-2016-097255. 28274916

[151] Malone S, Hughes B, Doran DA, Collins K, Gabbett TJ. Can the workload–injury relationship be moderated by improved strength, speed and repeated-sprint qualities? J Sci Med Sport. 2019; 22(1):29–34. doi: 10.1016/j.jsams.2018.01.010. 30057364

[152] Hulin BT, Gabbett TJ. Indeed association does not equal prediction: the never-ending search for the perfect acute: chronic workload ratio. Br J Sports Med. 2019; 51(1):144–145. doi: 10.1136/bjsports-2018-099448. 29886435

[153] Lathlean TJH, Newstead SV, Gastin PB. Elite Junior Australian Football Players With Impaired Wellness Are at Increased Injury Risk at High Loads. Sports Health. 2023; 15(2):218–226. doi: 10.1177/19417381221087245. 35524427 PMC9951000

[154] Rabbani A, et al. Match Fatigue Time-Course Assessment Over Four Days: Usefulness of the Hooper Index and Heart Rate Variability in Professional Soccer Players. Front Physiol. 2019; 10:109. 30837890 10.3389/fphys.2019.00109 PMC6390199

[155] Ullah S, Gabbett TJ, Finch CF. Statistical modelling for recurrent events: an application to sports injuries. Br J Sports Med. 2014; 48(17):1287–1293. doi: 10.1136/bjsports-2011-090803. 22872683 PMC4145455

[156] Silva JR, Rumpf MC, Hertzog M, et al. Acute and residual soccer match-related fatigue: a systematic review and meta-analysis. Sports Med. 2018; 48:539–583. doi: 10.1007/s40279-017-0798-8. 29098658

[157] Manuel Clemente F, Pillitteri G, Palucci Vieira LH, Rabbani A, Zmijewski P, Beato M. Balancing the load: A narrative review with methodological implications of compensatory training strategies for non-starting soccer players. Biol Sport. 2024; 41(4):173–185. doi: 10.5114/biolsport.2024.139071. 39416502 PMC11475008

[158] Riboli A, et al. Area per player in small-sided games to replicate the external load and estimated physiological match demands in elite soccer players. PLOS One. 2020; 15(9):e0229194. 32966305 10.1371/journal.pone.0229194 PMC7510966

[159] Riboli A, Coratella G, Rampichini S, Cé E, Esposito F. The distribution of match activities relative to the maximal intensities in elite soccer players: implications for practice. Res Sports Med. 2022; 30(5):463–474. doi: 10.1371/journal.pone.0229194. 33657944

[160] Riboli A, Semeria M, Coratella G, Esposito F. Effect of formation, ball in play and ball possession on peak demands in elite soccer. Biol Sport. 2021; 38(2):195–205. doi: 10.5114/biolsport.2020.98450. 34079164 PMC8139352

